# Multiomics integrative analysis identifies *APOE* allele-specific blood biomarkers associated to Alzheimer’s disease etiopathogenesis

**DOI:** 10.18632/aging.202950

**Published:** 2021-04-12

**Authors:** Laura Madrid, Sonia Moreno-Grau, Shahzad Ahmad, Antonio González-Pérez, Itziar de Rojas, Rui Xia, Pamela V. Martino Adami, Pablo García-González, Luca Kleineidam, Qiong Yang, Vincent Damotte, Joshua C. Bis, Fuensanta Noguera-Perea, Céline Bellenguez, Xueqiu Jian, Juan Marín-Muñoz, Benjamin Grenier-Boley, Adela Orellana, M. Arfan Ikram, Philippe Amouyel, Claudia L. Satizabal, Alzheimer’s Disease Neuroimaging Initiative (ADNI)*, EADI consortium, CHARGE consortium, GERAD consortium, GR@ACE/DEGESCO consortium, Luis Miguel Real, Carmen Antúnez-Almagro, Anita DeStefano, Alfredo Cabrera-Socorro, Rebecca Sims, Cornelia M. Van Duijn, Eric Boerwinkle, Alfredo Ramírez, Myriam Fornage, Jean-Charles Lambert, Julie Williams, Sudha Seshadri, ADAPTED consortium, Janina S. Ried, Agustín Ruiz, Maria Eugenia Saez

**Affiliations:** 1 Andalusion Bioiformatics Research Centre (CAEBi), Sevilla, Spain; 2 Research Center and Memory Clinic Fundació ACE, Institut Català de Neurociències Aplicades, Universitat Internacional de Catalunya, Barcelona, Spain; 3 CIBERNED, Network Center for Biomedical Research in Neurodegenerative Diseases, National Institute of Health Carlos III, Madrid, Spain; 4 Department of Epidemiology, Erasmus Medical Centre, Rotterdam, The Netherlands; 5 Institute of Molecular Medicine and Human Genetics Center, University of Texas Health Science Center at Houston, Houston, TX 77030, USA; 6 Division of Neurogenetics and Molecular Psychiatry, Department of Psychiatry and Psychotherapy, Medical Faculty, University of Cologne, Cologne, Germany; 7 Department of Neurodegenerative Diseases and Geriatric Psychiatry, University Hospital Bonn, Bonn, Germany; 8 German Center for Neurodegenerative Diseases (DZNE), Bonn, Germany; 9 Department of Biostatistics, Boston University School of Public Health, Boston, MA 02118, USA; 10 University Lille, Inserm, CHU Lille, Institut Pasteur de Lille, U1167-RID-AGE-Facteurs de Risque Et Déterminants Moléculaires des Maladies Liées au Vieillissement, Lille, France; 11 Cardiovascular Health Research Unit, Department of Medicine, University of Washington, Seattle, WA 98195, USA; 12 Unidad de Demencias, Hospital Clínico Universitario Virgen de la Arrixaca, Carretera de Madrid-Cartagena s/n, 30120 El Palmar, Murcia, España; 13 Glenn Biggs Institute for Alzheimer's and Neurodegenerative Diseases, UT Health San Antonio, San Antonio, TX 78229, USA; 14 Department of Neurology, Boston University School of Medicine, Boston, MA 02118, USA; 15 Unit of Infectious Diseases and Microbiology, Hospital Universitario de Valme, Sevilla, Spain; 16 Department of Surgery, Biochemistry and Immunology, University of Malaga, Spain; 17 Janssen Research and Development, a Division of Janssen Pharmaceutica N.V., Beerse, Belgium; 18 Division of Psychological Medicine and Clinical Neuroscience, School of Medicine, Cardiff University, Cardiff, UK; 19 Department of Epidemiology, Human Genetics, and Environmental Sciences, School of Public Health, The University of Texas Health Science Center at Houston, Houston, TX 77030, USA; 20 UKDRI@Cardiff, School of Medicine, Cardiff University, Cardiff, UK; 21 AbbVie Deutschland GmbH & Co. KG, Genomics Research Center, Knollstrasse, Ludwigshafen, Germany

**Keywords:** Alzheimer’s disease, APOE, integrative analysis, biomarkers

## Abstract

Alzheimer’s disease (AD) is the most common form of dementia, currently affecting 35 million people worldwide. Apolipoprotein E (APOE) ε4 allele is the major risk factor for sporadic, late-onset AD (LOAD), which comprises over 95% of AD cases, increasing the risk of AD 4-12 fold. Despite this, the role of APOE in AD pathogenesis is still a mystery. Aiming for a better understanding of APOE-specific effects, the ADAPTED consortium analysed and integrated publicly available data of multiple OMICS technologies from both plasma and brain stratified by *APOE* haplotype (*APOE2, APOE3* and *APOE4*). Combining genome-wide association studies (GWAS) with differential mRNA and protein expression analyses and single-nuclei transcriptomics, we identified genes and pathways contributing to AD in both APOE dependent and independent fashion. Interestingly, we characterised a set of biomarkers showing plasma and brain consistent protein profiles and opposite trends in *APOE2* and *APOE4* AD cases that could constitute screening tools for a disease that lacks specific blood biomarkers. Beside the identification of APOE-specific signatures, our findings advocate that this novel approach, based on the concordance across OMIC layers and tissues, is an effective strategy for overcoming the limitations of often underpowered single-OMICS studies.

## INTRODUCTION

Non-Mendelian Alzheimer’s disease (AD) has become the paradigm of a complex disease for which a major genetic determinant is known, the *APOE* locus. Three linkage studies published in 1993 pointed to the *APOE* region at 19q13 as a risk locus for late onset familial AD [1, 2], and even common sporadic late-onset AD (LOAD) [3]. Shortly after, researchers around the world confirmed the association of *APOE* gene with diverse forms of the disease and its association with other dementias.

The *APOE* gene encodes a lipoprotein firstly identified in the 1970s among patients with familial hypercholesterolemia type III [4, 5]. The protein has three major isoforms depending on the combination of two polymorphisms located at positions 112 (rs429358 (C > T)) and 158 (rs7412 (C > T). The most common isoform, *APOE*3, has a cysteine at position 112 and an arginine at position 158, whereas *APOE*2, the least common isoform, has a cysteine at both positions, and the AD risk allele *APOE*4 has an arginine at both positions [6–8]. These aminoacidic substitutions result in a conformational change that brings together the N-terminal and C-terminal domains in *APOE*4, which are normally separated in *APOE*2 and *APOE*3 isoforms. The consequences in downstream signaling of this conformational shift in the *APOE*4 isoform are still unknown. In fact, it is not even clear if the *APOE*4 is a gain or loss of function mutation despite extensive research in the field [9]. What is already known is that having a single *APOE*4 allele increases risk 2- to 4-fold and having two *APOE*4 alleles increases risk about 8- to 12-fold, although risk varies according to genetic background and sex [10].

In the last years, genome-wide association studies (GWAS) have contributed a number of Alzheimer’s disease associated low penetrance genes, including *ABCA7, ABI3, ACE, AC074212.3, ADAM10, ADAMTS1, ADAMTS4, ALPK2, ANKDR31, APH1B, ATP5H, BIN1, BZRAP1-AS1, CASS4, CD2AP, CD33, CELF1-MADD, CLNK, CLU, CNTNAP2, CR1, DSG2, ECHDC3, EPHA1, FERMT2, HESX1, HLA-DRB5–HLA-DRB1, HS3ST1, KAT8, IQCK, INPP5D, NME8, NYAP1, MS4A* gene cluster*, NDUFAF6, OARD1, PICALM, PLCG2, PTK2B, SCIMP, SLC24A4, SORL1, SPI1, TREM2, WWOX, ZCWPW1* [11–14]. Some reports have performed stratified analyses based on the presence or absence of the *APOE*4 allele, identifying some additional genes with effect in *APOE*4 carriers (such as *ISYNA1, CUGBP2)* or in individuals lacking the E4 allele *(AC099552, GPAA1, MAPT, NSF, TREM2*) [15–18].

One aim of the ADAPTED consortium is to identify specific *APOE* signatures associated with the different *APOE* isoforms. We describe for the first time herewith a comprehensive integration of genomic, transcriptomic and proteomic data stratified by the three major *APOE* haplotypes.

## RESULTS

### GWAS data: SNP-level analysis

**Figure 1:**
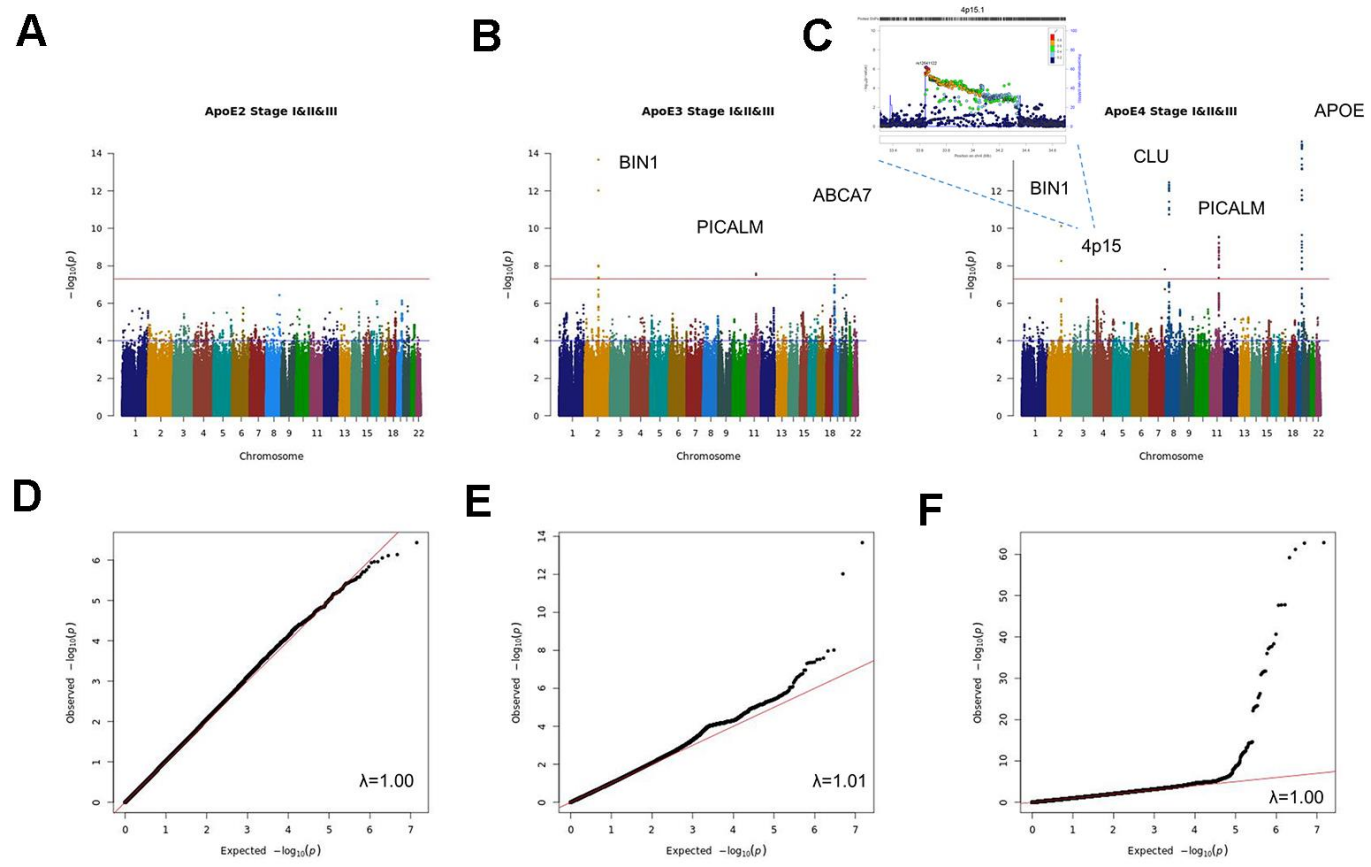
Manhattan and QQ plots of stage I+II+III APOE2 (**A**, **D**), APOE3 (**B**, **E**) and APOE4 (**C**, **F**) stratified meta-analysis.

The combined analysis of the three stages (stage I+II+III) (Figure 1 and Supplementary Tables 1–3 and Supplementary Figures 1–3), identified genome-wide significant signals (p<5x10^-8^) for *APOE*, *BIN1, CLU, CNTNAP2* and *PICALM* in the *APOE4* stratum; suggestive signals (p<10^-5^) in this analysis include a 1.4Mb intergenic region on 4p15. (from 33.3Mb to 34.7 Mb, hg19) with lowest p value for the SNP rs12641122 (p=6.28x10^-7^), a 4.5Kb intergenic region on 4q35.2 or the *KCNQ3* gene among others. In the *APOE3* stratum, *ABCA7, BIN1* and *PICALM* passed the genome-wide significance threshold, with suggestive signals for the *HLA-DQ/HLA-DR loci, CTNND2, FBN1, WLS* or *CSTF1* genes among others. By contrast, no genome wide significant SNPs were found in the *APOE*2 stratum, nor any known AD gene among suggestive signals.

An additional validation of stage I+II+III findings was performed using the EADI population (stage IV), where only the *APOE* locus in the *APOE4* stratum reached the genome wide significance threshold (Supplementary Figure 4). In the combined analysis of Stage I+II+III and Stage IV results, *ABCA7, BIN1* and *PICALM* in the *APOE3* stratum and *APOE, BIN1, CLU* and *PICALM* in the *APOE4* stratum reached genome-wide significance (Supplementary Tables 4–6 and Supplementary Figure 5).

Sex stratified meta-analysis (Supplementary Tables 7, 8 and Supplementary Figure 6) identified genome-wide significant signals for *BIN1* and *APOE* as well as suggestive signals for *PICALM, MYLK, SOX5* and *SCEL* in the female population. By contrast in males, only suggestive signals for *BIN1*, *APOE*, *ZCCHC2*, the *ABI3BP/IMPG2* locus, *ESRRB* and the 19q13.4 leukocyte receptor cluster were identified. Stratified analysis by sex and *APOE* (Supplementary Tables 9–14 and Supplementary Figure 7), yielded genome-wide significant signals for *APOE* in the *APOE*4 stratum in both sexes and for a 400kb 13q31.3 intergenic region containing the Ubiquitin Specific Peptidase 7 (Herpes Virus-Associated) (*USP7*) pseudogene (RP11-464I4.1) for *APOE3* males. Among *APOE*4 males, we found association with AD for a large region of 1.9Mb on 3q12.1 comprising the genes *CMSS1, COL8A1, FILIP1L, MIR548G, RPL24* and, in females, a 1.5Mb region on 2q33.2 comprising the *ABI3* homologue *ABI2, CARF, CYP20A1, FAM117B, FZD7, ICA1L, NBEAL1, RAPH1 and WDR12* genes.

### GWAS data: gene-level analysis

Genetic marker-level results were summarized into a single measure of association for each gene. Then, association results from the combined stage I, II and III meta-analysis were used to estimate gene-wide statistics for all genes in each one of the three *APOE* strata (Supplementary Tables 15–17). Per stratum, genes were ranked in ascending order according to lowest to highest p values derived from the mean *χ*2 statistics implemented in MAGMA (Table 1). Among previously reported AD genes, *APOE* was the highest ranked in both *APOE2* and *APOE4* carriers (ranks 26 and 3 respectively), whereas *BIN1* was ranked first in the *APOE*3 stratum (Supplementary Table 18). Known AD genes were ranked worst in the *APOE*2 stratum than in the others, with the complement receptor 1 (*CR1*), ranked in position 1292, being the second most relevant of these genes among *APOE*2 carriers after *APOE*.

**Table 1:** GWAS analysis: gene level data (MAGMA results) by *APOE stratum*.

**Rank E2**	**HUGO**	**N_SNPS_**	**N_PARAM_**	**Z_STAT_**	**P_Joint_**	**P_SNPwise (mean)_**	**P_SNPwise (top)_**
1	RNF152	609	65	4.62	1.90E-06	4.59E-07	4.63E-04
2	DUOX2	150	26	3.97	3.53E-05	4.72E-06	5.12E-03
3	METRN	140	18	3.88	5.29E-05	2.41E-05	9.43E-04
4	WDR24	197	18	3.83	6.31E-05	2.71E-05	1.21E-03
5	FBXL16	196	18	3.84	6.03E-05	2.85E-05	1.11E-03
6	FAM173A	120	17	3.83	6.29E-05	2.99E-05	1.14E-03
7	JMJD8	193	18	3.79	7.59E-05	3.34E-05	1.32E-03
8	CCDC78	115	17	3.81	6.99E-05	3.68E-05	1.14E-03
9	DUOXA2	145	23	3.63	1.41E-04	3.68E-05	6.05E-03
10	HAGHL	107	15	3.83	6.43E-05	3.88E-05	9.08E-04
11	NARFL	105	15	3.83	6.51E-05	4.00E-05	8.92E-04
12	STUB1	193	18	3.72	1.01E-04	4.61E-05	1.56E-03
13	ABCB4	344	35	3.89	5.11E-05	5.27E-05	5.59E-03
14	APOC1	209	32	3.42	3.16E-04	5.40E-05	4.13E-02
15	IRGC	300	45	4.55	2.72E-06	5.52E-05	3.36E-05
**Rank E3**	**HUGO**	**N_SNPS_**	**N_PARAM_**	**Z_STAT_**	**P_Joint_**	**P_SNPwise (mean)_**	**P_SNPwise (top)_**
1	BIN1	633	70	7.02	1.15E-12	8.93E-07	3.51E-12
2	FBN1	533	41	4.71	1.27E-06	4.82E-06	2.76E-04
3	WNT3	239	38	4.69	1.35E-06	6.85E-06	1.26E-04
4	CLEC4M	424	68	4.07	2.35E-05	1.35E-05	1.46E-03
5	NSF	144	22	4.76	9.48E-07	2.88E-05	3.07E-05
6	CASS4	349	50	5.00	2.80E-07	3.43E-05	4.58E-05
7	APP	998	99	3.96	3.75E-05	3.91E-05	3.43E-03
8	GPR27	306	47	3.21	6.62E-04	4.63E-05	6.29E-02
9	CRHR1	1261	23	4.07	2.39E-05	5.46E-05	2.55E-03
10	CD209	482	71	3.80	7.36E-05	6.93E-05	1.55E-03
11	SPPL2C	556	19	4.03	2.83E-05	7.77E-05	1.68E-03
12	KANSL1	978	20	4.01	2.98E-05	7.79E-05	1.44E-03
13	LRRC37A	115	6	4.01	3.09E-05	7.97E-05	4.41E-04
14	STH	460	17	3.94	4.00E-05	8.35E-05	2.88E-03
15	EIF4E3	516	83	3.01	1.29E-03	9.34E-05	1.41E-01
**Rank E4**	**HUGO**	**N_SNPS_**	**N_PARAM_**	**Z_STAT_**	**P_Joint_**	**P_SNPwise (mean)_**	**P_SNPwise (top)_**
1	TOMM40	293	44	14.90	1.59E-50	1.50E-26	1.00E-50
2	APOC1	247	38	15.05	1.71E-51	3.20E-26	1.00E-50
3	APOE	270	40	14.82	5.36E-50	2.83E-24	1.00E-50
4	PVRL2	361	51	14.31	8.88E-47	1.17E-23	1.00E-50
5	APOC4	241	29	11.30	6.96E-30	1.60E-14	2.52E-33
6	APOC2	232	27	13.20	4.31E-40	8.54E-13	1.00E-50
7	CLPTM1	267	34	12.78	1.06E-37	3.43E-11	5.65E-46
8	CLU	351	46	7.60	1.47E-14	5.85E-10	4.89E-13
9	SCARA3	426	51	7.08	7.06E-13	7.15E-07	3.53E-13
10	PICALM	555	43	5.54	1.49E-08	8.39E-07	9.65E-08
11	AKAP2	601	85	4.12	1.86E-05	2.27E-06	5.58E-03
12	PALM2-AKAP2	1542	159	3.61	1.52E-04	3.72E-05	1.46E-02
13	IL6	414	48	3.72	1.01E-04	4.36E-05	7.17E-03
14	EPHX2	467	56	4.23	1.15E-05	5.26E-05	2.17E-04
15	BIN1	635	70	5.70	5.95E-09	5.72E-05	1.11E-08

### Differential expression analysis

**Figure 2:**
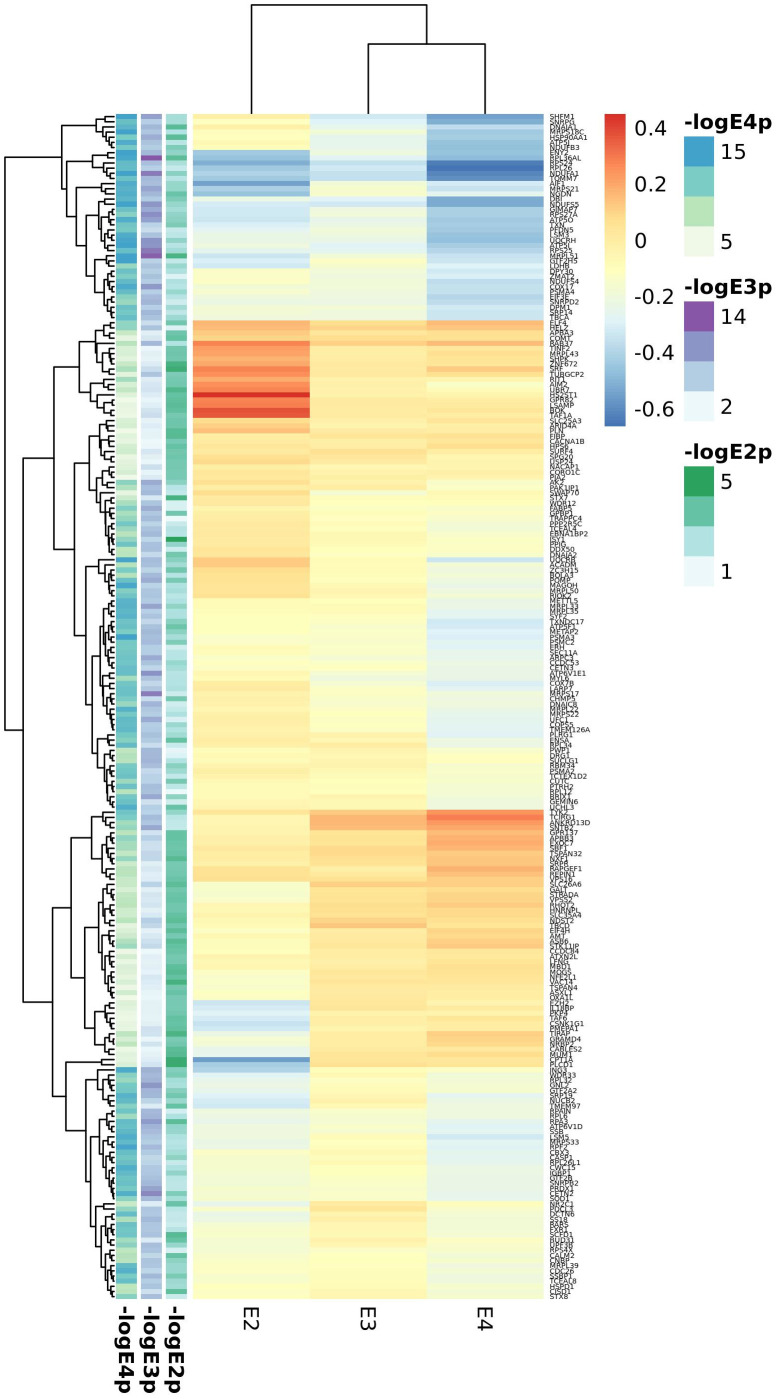
**Top candidates from DE analysis in blood datasets.**

Blood APOE stratified DE meta-analysis between AD cases and controls (Supplementary Tables 19–21) included the ADNI and ADDN datasets. In the *APOE*2 stratum we identified only two upregulated (*ISY1* and *SRF)* and two downregulated *(CPT1A, PLCD1)* genes below the FDR <0.05 threshold, clearly differing from expression profiles in *APOE3* and *APOE4* carriers (Figure 2, top 100 genes from each stratum). By contrast, *APOE3* and *APOE4* stratified analyses identified 1,692 and 3,293 DE genes respectively. Among genes differentially expressed in *APOE4* cases versus controls we observed an over-representation of mitochondrial genes, most of them involved in the oxidative phosphorylation pathway. However, several genes from this pathway were differentially expressed in all strata but with opposite expression profiles, such as the electron transport chain genes *ATP5F1*, *UQCRB* or *NDUFB3* upregulated in *APOE2* cases but downregulated in *APOE4* cases when compared to controls of the same haplotype. *APOE3* genes were mainly cytoplasmatic genes involved in RNA metabolism.

**Figure 3:**
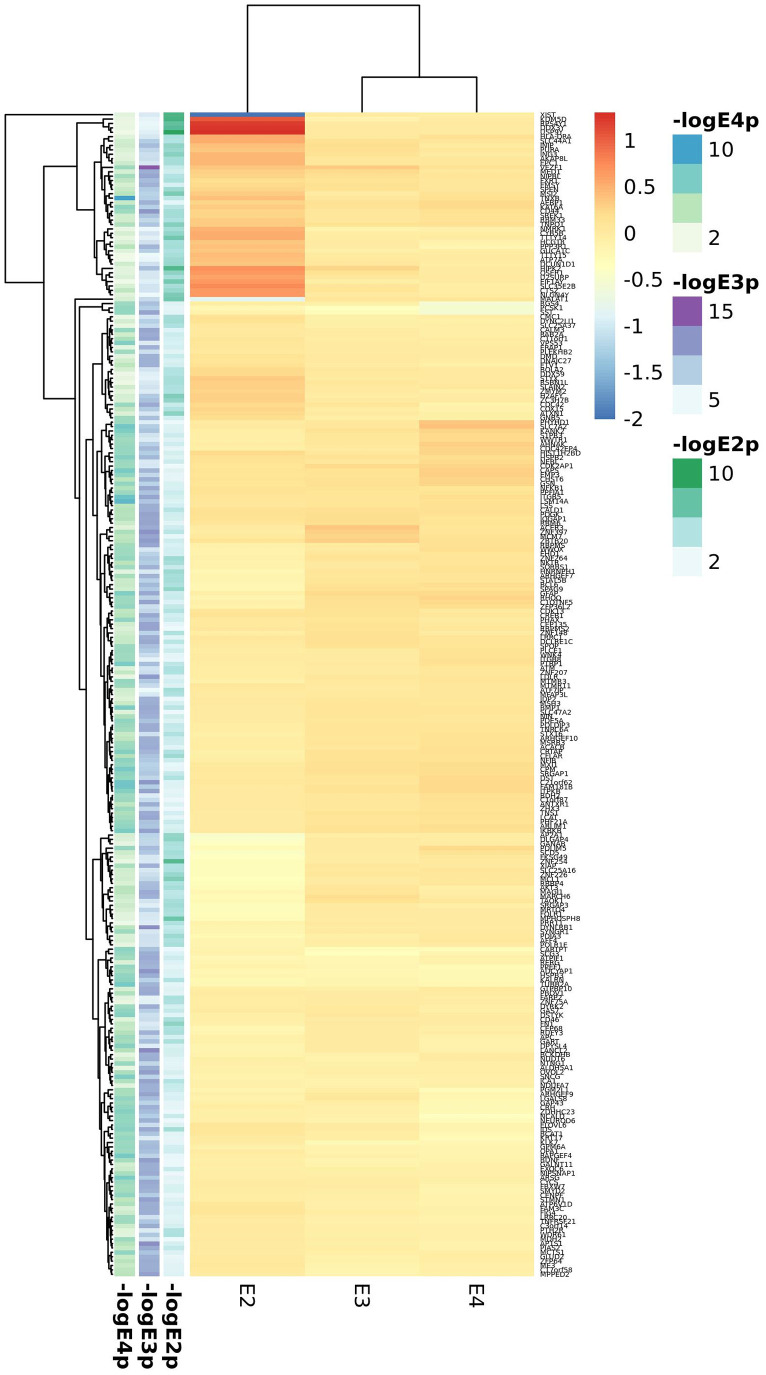
**Top candidates from DE analysis in cortex datasets.**

Cortex APOE-stratified DE included the MAYO, ROSMAP, MSBB, GSE15222 and GSE48350 studies. Meta-analysis of cortex datasets resulted in 518, 7714 and 1717 statistically significant genes (FDR<0.05) for the *APOE2*, *APOE3* and *APOE4* strata respectively (Supplementary Tables 22–24). As opposed to blood analyses, the overall picture is of enhanced gene expression in AD in all strata, but more pronounced in *APOE2* except for *XIST*, strongly downregulated in AD *APOE2* subjects (Figure 3, top 100 genes from each stratum). The heparan sulfate proteoglycan *CD44* and the heparan sulfate lysosomal degradation enzyme *IDS* encoding genes were differentially expressed in all strata, with *CD44* strongly upregulated in *APOE2* cases and *IDS* downregulated in *APOE4* cases. *APOE2* specific genes were mostly nuclear genes involved in primary metabolic processes, as well as some apoptosis related genes (*CFLAR, ATM, MCL1, AKT3 and CTSZ*), all of them downregulated in AD cases but *CTSZ*, with higher expression in AD cases than in controls. *APOE*3 and *APOE*4 candidate genes were mainly expressed in the cytoplasm. In all strata, we identified genes involved in neuronal development (such as *GFAP, BDNF* or *CDC42*), especially in the *APOE3* stratum. For both *APOE2* and *APOE4* strata, genes involved in vesicle mediated transport were identified, with key genes such as *PCSK1, SYTL2* or *SVOP* downregulated in *APOE4* cases.

### Robust rank aggregation analysis

Integrative analysis was performed independently to include either blood or cortex APOE-stratified DE rankings. Thus, we combined meta-GWAS stage I-III gene-level results with blood meta-GWES results (Supplementary Tables 25–27) or with cortex meta-GWES results respectively (Supplementary Tables 28–30).

In blood 275, 284 and 278 genes passed the FDR threshold in the *APOE*2, *APOE*3 and *APOE*4 strata respectively with 15 common genes, associated with AD irrespective of the APOE haplotype, which include *APOC1, CLPTM1, DNAJA1, ING3, LARP7, NGDN, RPA3, RPL36AL, RPS24, SOD1, SRP19*, and four mitochondrial proteins (*ATP5F1, ATP6V1D, MRPL51* and *UQCRH*). The list of APOE2 specific genes is the largest one (241 genes) and include mitochondrial transporters such as *SLC25A3* and *SLC25A4*. *APOE3* specific signatures included *MAPT* and *APP* genes, along with other neuronal genes such as the cholinergic receptors *CHRNA10* and *CHRNA2*. APOE4 specific genes included known AD genes involved in vesicle transport such as *CLU* and *SORL1*; SORL1 has been shown to regulate IL6 levels, also identified among *APOE4* specific signatures. We found however a large overlap among significant genes for the *APOE*3 and *APOE*4 strata (n=51) including *BIN1, MS4A4A, MS4A6A, PICALM* and *SLC24A4* AD genes and a good number of ribosomal and electron transport mitochondrial proteins (*ATP5I, ATP5O, ATP6V1E1, COX17, MRPL27, MRPL33, MRPL35, MRPS17, MRPS21, UQCRB*). *APOE*2 and *APOE*3 shared eleven genes (*ACADM, AK2, ENSA, GPR132, GPR137, HSP90AA1, MCL1, NXF1, TAF1C, UBA7, ZC3H15*) whereas the overlap between E2 and E4 was the smallest with eight proteins (*KTN1, NDST2, POLR1D, RHOT2, STK17B, TOMM40, TSPAN32, UCHL3*). At the pathway level, we observed a lower overlap between *APOE* strata, with only three shared mitochondrial GO categories among the three haplotypes, and little or none overlap between *APOE*2 and *APOE*3 or *APOE*2 and *APOE*4 (Supplementary Tables 31–33 and Supplementary Figure 8). In contrast, there was considerable overlap between *APOE*3 and *APOE*4 which includes *mitochondria* biology, *secretory* vesicles and *antigen processing and presentation* functions.

In cortex, we found 376, 399 and 366 significant genes (FDR<0.05) for the *APOE*2, *APOE3* and *APOE*4 strata respectively. Seven common AD candidate genes (*APOC1, APOC2, CD44, CDC42, CLPTM1, DST, PGM2L1*) were significant in all three strata; of them, *APOC1* and *CLPTM1* were also associated in the three strata in blood. In this analysis, the shortest list of *APOE*-specific genes was found in the *APOE4* stratum, which showed the largest overlap with the *APOE3* stratum; among the 45 significant genes shared by these strata, we identified several AD genes, including *BIN1, MS4A4A, MS4A6A, PICALM and RIN3* in accordance with blood results. The *CR1* gene is included among the 19 top genes from the *APOE*2 and *APOE*3 strata (*ATPIF1, CACNB2, CDC27, CFLAR, COX15, CR1, DCLK1, GOSR2, KANSL1, KLF12, MAPT, MCL1, NSF, POGK, RUFY3, SCD5, SORBS1, SPEN, TTN*), whereas *APOE*, *TOMM40, SLC24A4* or *WWOX* were included among common genes for the *APOE*2 and *APOE*4 strata (21 genes: *AHNAK, APOE, ARNT, CRTAP, FBXL16, GART, KALRN, KAT6A, MTMR11, OPA1, PDLIM5, PPFIA1, PURA, RBMS2, SLC24A4, SRGAP1, TOMM40, TSPAN14, UBE2F, WWOX, ZNF264*). Enrichment analysis also identified both common and exclusive pathways. Common pathways for AD irrespective of the *APOE* haplotype were related to *adhesion*, *neuronal development*, *differentiation,* and lipoprotein metabolism; diverse signals related to *neuronal death* are also present in all three strata. Again, we observed larger overlap between *APOE*3 and *APOE*4 pathways (*glial cell differentiation and activation, immunological, lipid metabolism, cardiovascular system development and heart function*) than for *APOE*2 and *APOE*3 (which includes *axonogenesis*) or *APOE*2 and *APOE*4 (mainly *phospholipid* and *lipoprotein* metabolism due to *APOE, APOC1* and *APOC2* genes). (Supplementary Tables 34–36 and Supplementary Figure 9). We observed that *APOE*2 exclusive pathways include *chromatin regulation* and *telomere maintenance* related processes. The *APOE*3 strata showed the largest number of significant enrichments, but most of them showed a similar annotation in *APOE*2, or, more frequently, in *APOE*4 strata, with the exception of *antigen processing and presentation*, *IFNG signalling*, *astrocyte development and activation* and *myelin sheath*. In *APOE*4 *macrophage activation*, *fructose metabolism*, *vitamin D mediated inflammation*, *inositol phosphate metabolism* and *cholesterol efflux* were the most relevant pathways. *Clathrin vesicles*, *amyloid biology*, *inflammatory and immune response* and *glial cell development and differentiation* appear as the most relevant categories shared by *APOE*3 and *APOE*4 strata.

**Figure 4:**
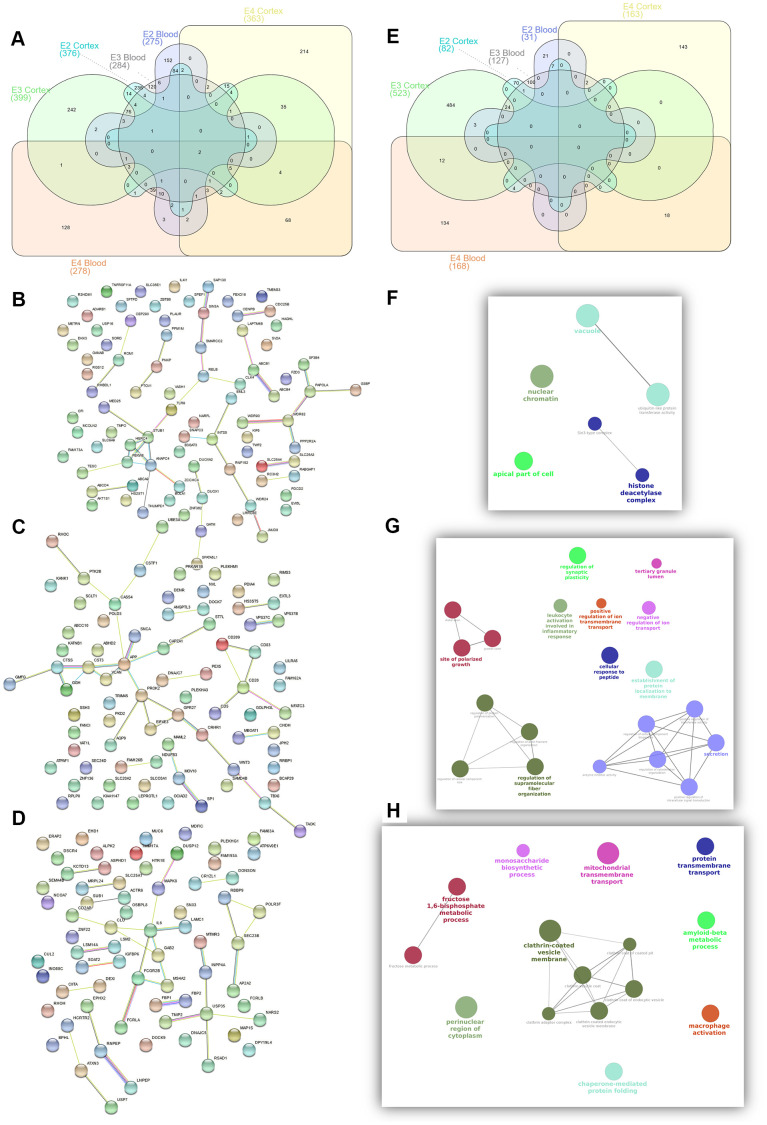
Summary of candidate genes (**A**–**D**) and pathways (**E**–**H**) from APOE2 (**B**, **F**), APOE3 (**C**, **G**) and APOE4 (**D**, **H**) common candidates from Blood and Cortex RRA analyses.

**Figure 5:**
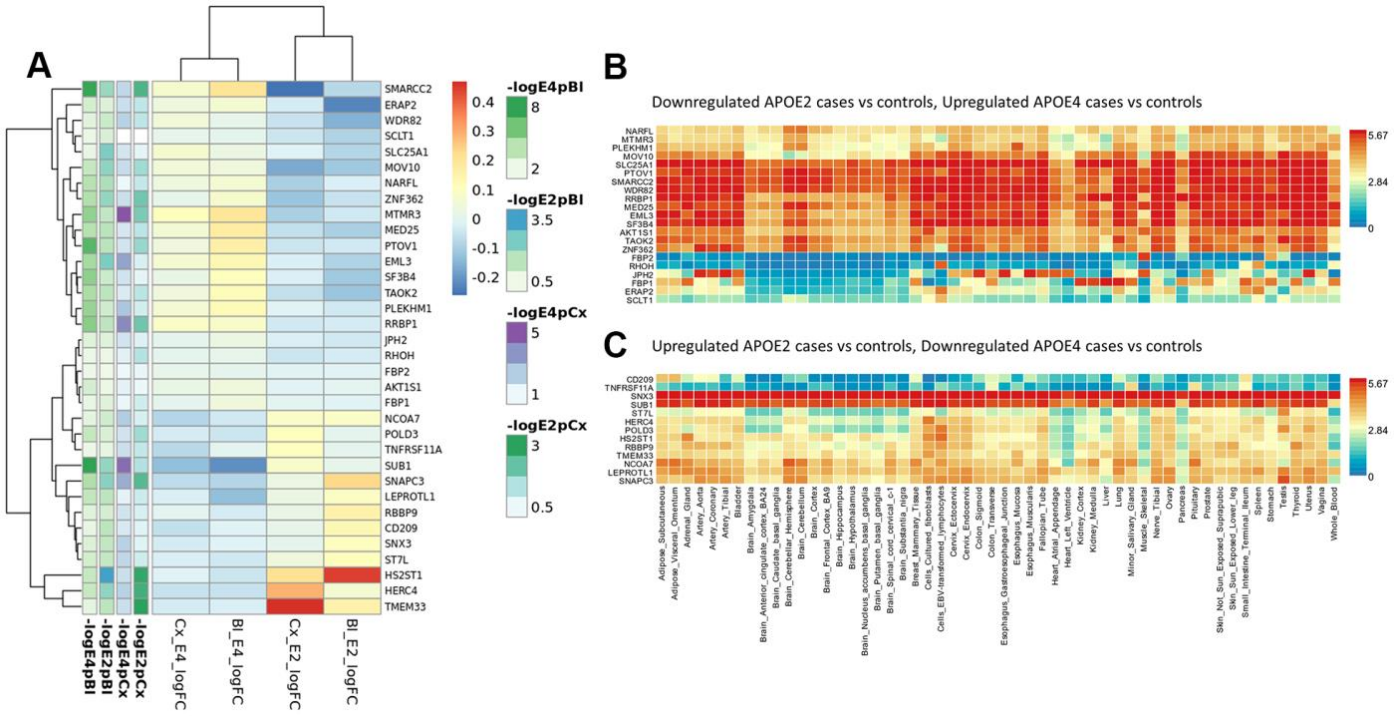
**Blood and cortex biomarkers showing opposite profiles in APOE2 and APOE4 strata.** (**A**) Meta-analysis logFCs from case-control DE analysis in blood and cortex (APOE2 and APOE4 strata); (**B**) average expression of genes downregulated in APOE2 and upregulated in APOE4 cases by normal tissue (GTEx repository); (**C**) average expression of genes upregulated in APOE2 and downregulated in APOE4 cases by normal tissue (GTEx repository).

To identify relevant candidate blood biomarkers tracking brain changes in AD pathology we compared blood and cortex analyses (Figure 4). We identified 68 genes in common for the *APOE*4 stratum, including *CLU, CD2AP, IL6, MS4A2, SLC25A1* or *INNPP4A* (Figure 4C). In *APOE*3, 76 common genes were found, including *APP, AQP9, ATPAF, CD209, LILRA5, NDUFB3* or *PTK2B* (Figure 4D). Finally, in the *APOE2* stratum, we identified 84 common genes including several ABC receptors (*ABCA9, ABCB1, ABCB4, ABCD4*), solute carrier molecules (*SLC25A3, SLC25A4, SLC35E1, SLC9A9*), *TLR9* or *IL4I1* (Figure 4B). Overlap between *APOE* strata-specific pathways from blood and cortex showed 6 common pathways for *APOE*2 (Figure 4F), half of them related to chromatin regulation, 24 common pathways for *APOE*3 (secretion, regulation of supramolecular fiber organization, site of polarized growth and leukocyte activation involved in inflammatory response, Figure 4G) and 18 shared pathways for APOE4, including clathrin coated vesicles, amyloid-beta processing, mitochondrial transmembrane transport, macrophage activation and monosaccharides and fructose metabolism (Figure 4H). We followed up genes with concordant profiles in both blood and cortex (upregulated or downregulated in AD cases vs controls) and showing opposite profiles in *APOE2* and *APOE4,* which included 34 genes with overrepresentation of the *gluconeogenesis* and *fructose metabolic pathways* (*FBP1, FBP2, SLC25A1*) (Figure 5A). When compared with average expression in normal brains, *FBP1, FBP2, RHOH, JPH2, ERAp2* and *SCLT1* were upregulated in APOE4 cases when they are usually expressed at low levels, whereas, *SNX3* and *SUB1*, were downregulated in *APOE4* cases when they are expressed at very high levels in the normal brain according to GTEx (Figure 5B).

### Validation on proteomic datasets

We aimed at investigating if any of our candidate genes were detected and differentially expressed at the proteomic level using blood proteomics data from the ADDN study (931 proteins) and cortex proteomics from four independent datasets (BANNER, BLSA, MAYO and MSBB, 2,658 proteins).

Out of 737 RRA blood candidates, only 38 were present in the ADDN blood proteomic data (Supplementary Table 37 and Supplementary Figure 10). Among them, DE analyses between cases with controls, either overall or stratified by APOE haplotype, identified 8 differentially expressed genes in the unstratified analysis, 8 genes in the APOE4 stratum and 9 in the APOE3 stratum. We could confirm APOE allele-specific effects identified in the RRA analysis for the immune related proteins AIF1, METAP2, NCK1, PRDX1, PRKCZ, RPS27A in the APOE3 stratum, and FCGR2B and SEZ6L2 (involved in SNC development) in the APOE4 stratum. Overall, among these 38 RRA candidates, we identified a cluster of 11 overexpressed proteins in AD cases when compared to controls in the APOE3 stratum, but downregulated APOE4 AD cases including AIF1, APP, GDI2, HSP90AA1, METAP2, NACA, NCK1, PRDX1, RPS27A, SFTPD and UFC1 (Supplementary Figure 11); immunological functions associated to these proteins include leukocyte activation (APP, PRDX1, GDI2), Toll-like receptors (TLRs) cascade (APP, RPS27A, SFTPD) or phagocytosis (NCK1, HSP90AA1, SFTPD, AIF1) in line with our RRA findings.

In cortex, 234 out of 1,039 RRA candidates were present in the proteomics DE meta-analysis, 100 of them showing evidences of association (p<0.05) in at least one stratum or in the unstratified analysis (Supplementary Table 38 and Supplementary Figure 12). Of note, the largest differences between cases and controls were observed among *APOE4* carriers, confirming at the proteomic level the role of *APOE4* RRA candidates involved in *neurogenesis* (DPYSL4, EHD1, GABRB3, MAPK8, UNC13A), or more specifically, in *glial cell differentiation* (CLU, GAP43, GFAP, GSN). Among *APOE3* candidates, we confirmed candidates involved in *neurotransmission* such as RPH3A PTK2B, ALDH5A1, GABRA2 and APP (the later upregulated in all strata) and genes from the electron transport chain (ALDH5A1, NDUFA7, NDUFB3). Confirmed *APOE2* candidates included the choline transporter SLC44A1, involved in *myelin production*, and the myelin basic protein MBP; MAPT was upregulated in all strata but particularly in the *APOE2* stratum. We also confirmed the role of CDC42 and DST in all the strata, but we did not observe association of CD44 and PGM2L1 with AD in this analysis.

### Cell-type-specific expression profiles: cortex snRNAseq

**Figure 6:**
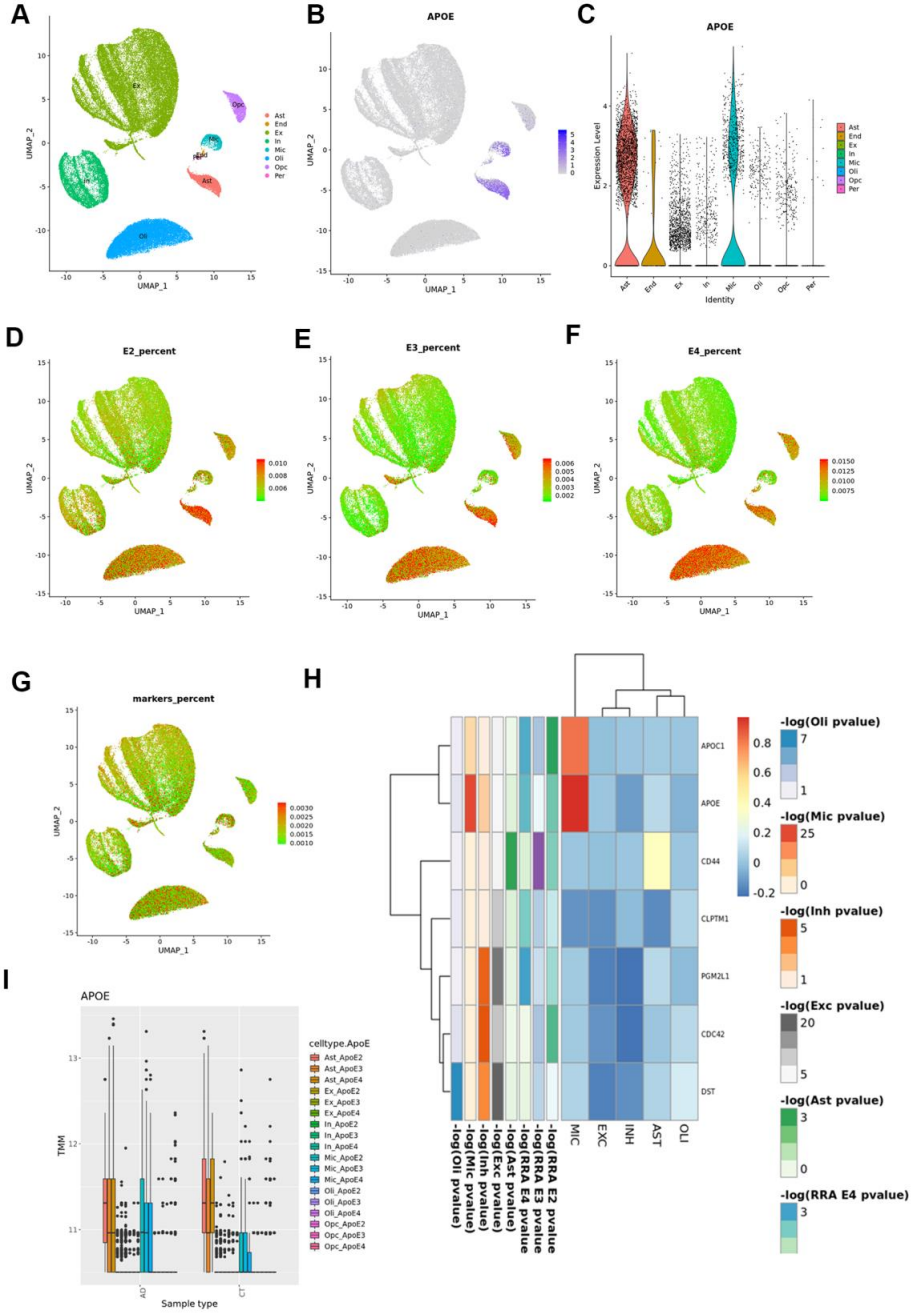
**Cortex snRNAseq data from the ROSMAP study.** (**A**) Cell clustering labelled by reported cell type; (**B**) APOE expression across cell types; (**C**) violin plot for APOE expression by cell type; (**D**) UMAP plot for average expression of cortex RRA APOE2 candidates by total gene expression; (**E**) UMAP plot for average expression of cortex RRA APOE3 candidates by total gene expression; (**F**) UMAP plot for average expression of cortex RRA APOE4 candidates by total gene expression; (**G**) UMAP plot for average expression of blood/cortex biomarkers by total gene expression; (**H**) expression by cell type of the seven genes in common in all the RRA analyses; (**I**) APOE expression by cell type and case status.

Since the enrichment analysis showed an over-representation of neuronal development related pathways in all strata, and of cells from the glial lineage in the *APOE3* and *APOE4* strata, we investigated which cerebral cell types our cortex RRA candidates were mainly expressed in, and which cell types showed largest differences between cases and controls using snRNAseq from the ROSMAP study (Figure 6). We dropped pericytes and endothelial cells from the differential expression analysis because of the low number of cells (≈100 cells, <0.3%).

*APOE* gene was mainly expressed in astrocytes and microglia (Figure 6). According to previous results, *APOE* is upregulated in microglia from AD subjects when compared with controls (overall and stratified by *APOE* genotypes). By contrast, in astrocytes we found higher *APOE* expression levels in cases than controls in the *APOE*3 stratum (logFC=0.34, p=1.56x10^-4^), but significant lower expression in *APOE*4 cases than in controls (logFC=-0.14, p=1.83x10^-2^, p_interaction_<10^-5^).

As reported in the original article [19], most neuronal genes were strongly differently expressed in AD cases versus controls. Furthermore, our analysis found this result was consistent irrespective of the *APOE* haplotype. Given that glial specific signals arose from *APOE3* and *APOE4* strata, we therefore primarily focused on RRA cortex candidates showing evidence of association with AD in any glial cell type (astrocytes, microglia, oligodendrocytes and oligodendrocyte precursors) within the same *APOE* stratum (Figure 6 and Supplementary Table 39 and Supplementary Figures 13–16). In fact, RRA candidates were mainly expressed in the glial lineage, showing a lineal decrease in expression from *APOE*2 to *APOE*4 in the astrocyte and microglia populations, and an increase in expression in the oligodendrocyte subpopulation (Figure 6). The seven genes in common in all the RRA analyses, were downregulated in all cell types except for *APOE* and *APOC1* in microglia, and *CD44* in astrocytes (Figure 6). In the stratified analysis, these 7 genes were predominantly downregulated in AD *APOE*3 carriers and upregulated among *APOE*4 AD cases when compared to controls, particularly *APOC1*, *DST* and *CD44* (Supplementary Figure 9). By stratum, *APOE*2 RRA cortex candidates were mostly upregulated in all cell types (Supplementary Figure 10), and in particular *FXR1* and *DNAJB1*, the latter only downregulated in microglia. *APOE*3 RRA candidates showed the largest differences between cases and controls in microglia cells, where *APOC1, ALDOA, RPLP0* and *DYNLRB1* were strongly upregulated whereas *ARL17B* was downregulated in AD cases Supplementary Figure 11). Almost all *APOE4* candidate genes were downregulated in both excitatory and inhibitory neurons and upregulated in the glial lineage, particularly *TMEM163* and *CPM* in microglia and *GFAP, PLCE1, CLU, CALN1, DLG2* and *PDE5A* in astrocytes Supplementary Figure 12); we also observed a strong downregulation of the Serine/Threonine Kinase 17b (*STK17B)*, involved in apoptosis and autophagy, in microglial cells of *APOE*4 cases.

## DISCUSSION

The ADAPTED consortium has performed a holistic approach analyzing and integrating diverse data sets from different OMICS technologies, including genomics, transcriptomics (bulk tissue and single cell) and proteomics collected from public repositories and other consortia, resulting in nearly sixty thousand samples analyzed. The novelty of our strategy relies on the use of a stratified approach for the three major *APOE* haplotypes, and the integration of these signals with a ranked-based algorithm which accommodates different kind of data, resulting in replicated signals at different levels. These signals have been further explored at the single-cell level, pointing to key cellular types for AD. Previous attempts for integrating different OMICS in AD were mainly focused on the identification of quantitative trait loci (QTLs) for mRNA levels, protein levels or epigenomic signatures by means of association analyses [20–23], in some cases stratified by *APOE* allele [24]. Other approaches involved the independent analysis of the different OMICS and selection of concordant genes [25] or the combination of human GWAS data with mouse transcriptomics [26]. Potential limitations of our study include reduced sample size in some of the datasets, especially for the A*POE2* stratum, and the use of unsigned methods (i.e. irrespective of the directionality of the expression profiles) for selecting candidate genes in expression datasets.

At the genome level, we were able to detect genome-wide significant signals for *ABCA7, BIN1* and *PICALM* in the *APOE3* stratum and for *APOE, BIN1, CLU* and PICALM in the *APOE4* stratum. We identified a novel candidate region for *APOE4* carriers on 4p15.1 (33.6Mb-34.3Mb), which, according to the GWAS catalogue (https://www.ebi.ac.uk/gwas/) has not been previously associated with AD, but with schizophrenia, total cholesterol change in response to fenofibrate in statin-treated type 2 diabetes, and *PCSK9* levels, a protease that binds to lipoprotein receptors promoting their degradation; a homozygous deletion overlapping this region has been described for the offspring of a consanguineous marriage between first cousins, with cognitive impairment and autistic-like behavior [27]. Sex-stratified analysis identified genome wide significant signals for *APOE* and *BIN1* only in females; this result is in agreement with the recent report from Fan et al., who described a genome-wide significant association for BIN1 only in females [28]. Further stratification of male and female populations by *APOE* haplotype identified a genome-wide significant intergenic region on 13q31.3 among *APOE3* males. This region has been associated with *TREM2* levels, circulating Interleukin-1-receptor antagonist levels and triglyceride change in response to fenofibrate in statin-treated type 2 diabetes. This region harbors a USP7 pseudogene (*RP11-464I4.1*) associated with herpesvirus. Interestingly, a potential role of herpes simplex virus infection in AD has recently been object of intense debate [29]. Despite the number of GWAS datasets collected, our study is still underpowered for detecting genuine *APOE* strata-specific signals with low effect sizes, but resulting gene-level statistics were instrumental to select those DE signals that better correlate with the disease at genetic level. This helps maximize high probable *loci* involved in the fundamental pathways involved in disease pathogenesis.

The genome-wide expression analysis was performed at two levels: blood and brain cortex. In blood, mitochondrial ribosomal genes and as well as those encoding proteins of the respiratory chain appeared downregulated in cases irrespectively of the *APOE* haplotype, but more pronounced among *APOE4* carriers. Mitochondria are crucial players of energy metabolism but are also the main source of Reactive Oxygen Species (ROS). Mitochondrial dysfunction has been proposed as the primary process triggering all the cascade of events that lead to sporadic late-onset AD. Although this hypothesis has not been confirmed, diverse mitochondrial functions were observed altered in AD and even MCI subjects, showing a significant increase of oxidative stress markers, such as lipid peroxidation and protein oxidation products [30–32]. We did not observe mitochondrial signatures at the whole cortex level, mostly enriched in activated genes from *neuronal, apoptosis, vesicle mediated transport* and *adhesion* related pathways, maybe because mitochondrial dysfunction has been reported to be limited to certain hippocampal and temporal cortex neurons [33, 34].

Integration of genome data with expression data at blood and cortex levels through the RRA algorithm, showed a larger overlap of genes and functions in *APOE3* and *APOE4* carriers than in *APOE2* carriers, which appears as a more distinct entity. In fact, we identified signatures for *chromatin remodeling* and regulation in this stratum at both brain and plasma levels, not observed in the other two strata. Common features of the disease to all three strata are related to *lipid metabolism* due to *APOE* (except for the *APOE3* carriers), *APOC1* and APOC2. A recent report has suggested that *APOC1* gene, located in the *APOE* locus, is an independent risk factor for AD, and that genetic variability in the region is associated with chromatin regulation [35].

AD cases in *APOE3* and *APOE4* share signaling pathways and functional categories previously reported by other groups such as *amyloid-beta formation* (*APOE, BIN1, CLU, PICALM*) *mitochondrial* physiology (including ATP5H, *NDUFS5, MRP* proteins*, SNCA, SOD1, SSBP1, SUCLG1 or UQCRH*), *vesicle* mediated transport (including *APOC1, APP, BIN1, C1QTNF5, CASS4, CDC42, LDLR, MAPT, PICALM* or *PTK2B*), *actin* organization (*ACTN1, ACTR2, AIF1, ANTXR1, CALD1, CAPZ1, CD2AP, DST, ITGB5, MACF1, MAPT, PALLD, RHOC …*) or *immunological* functions (*CCL5, CD209, CD44, CR1, IL6, LILRA5 or MS4A2* among others), but with specific gene signature (for example *IL6* in the *APOE4* stratum or *CD209* in the *APOE3* stratum). IL6 plays a critical role in inflammation as well as in neuroprotection through two different mechanisms. Anti-inflammatory effects are mediated by the classical signaling pathways, which involves the binding of IL6 to the membrane bound IL6 receptor (IL6R), whereas proinflammatory effects are mediated by soluble IL6R forms. Classical signaling occurs in microglia whereas trans-signaling is predominant in most neuronal types, astrocytes and oligodendrocytes [36]. Cross-talk between TREM2, CD33 and IL6 (among other ILs) regulating phagocytic capacity, a hallmark of AD among APOE4 carriers according to our results, has been reported in microglia cells [37]. Interestingly, IL6 is degraded by SORL1, encoded by another well-known AD gene [38]. CD209 is mainly expressed on the surface of dendritic cells, specialized antigen-presenting cells, where regulates DC adhesion, migration and triggering of immune response [39]. In conclusion, our results suggest that APOE-allele specific immunological checkpoints may exist in AD.

Although we have identified signatures of the nervous system development in all strata, they represent a largest proportion of relevant pathways in the *APOE3* stratum. In this stratum, enrichment analysis of RRA cortex candidates showed an over-representation of genes involved in cardiac development and function (*DLG1, JPH2* or *MEF2C* among others), supporting a cardiovascular etiology of dementia in this stratum. In line with this finding, we have recently reported a link between cardiac function and AD, that is mediated, at least in part, by *CFLAR* and caspase dependent mechanisms [40]. In fact, *CFLAR* and *CASP8* are both RRA cortex candidates in this stratum. Another example of the nervous-cardiac connection is *GFAP*, which participates in the control of heart rate and vascular resistance through the sympathetic nervous system (SNS), which controls heart rate and vascular resistance. We have observed an upregulation of GFAP protein in cortex of all AD cases irrespective of the *APOE* carrier status. *Macrophage activation* and *Fc gamma receptor mediated phagocytosis* appeared as the most exclusive pathways in the *APOE4* stratum. Phagocytosis (i.e. the engulfment and digestion of cellular debris) is critical for the degradation of infectious agents and senescent cells, playing a key role in tissue remodeling, immune response, and inflammation. Several Fc receptors (FcRs, *FCGR2B, FCRLA* and *FCRLB*) and downstream effectors receptor such as *CDC42, RHOH, RHOQ* and *RHOT2*, GTPases that regulate actin cytoskeleton, have been identified as *APOE4* RRA candidates. While FcRs are constitutively active for phagocytosis, the complement receptor (CR)-mediated phagocytosis is activated in presence of additional stimuli. An additional difference between FcR- and CR-mediated phagocytosis is that he former have a higher capacity for triggering the release of inflammatory mediators [41]. In fact, an enhanced release of inflammatory molecules such as IL-6, an *APOE4* RRA candidate, IL1β or TNFα has been observed in blood among *APOE4* carriers [42, 43] and in blood and brain humanized *APOE4* mice models [44–46] has been observed. In this study, we found that *CR-*related mechanisms were more relevant in *APOE2* and *APOE3* carriers, with CR1 and *ATP5F1* as RRA candidates in both strata.

Macrophages are also involved in the development of atherosclerotic plaques through the intracellular accumulation of lipids and the formation of foam cells, a process counterbalanced by cholesterol efflux, a mechanism identified as an APOE4 specific feature in our study. A key protein in this process seems to be AIF1, a pro-inflammatory molecule expressed primarily in the monocyte/macrophage lineage, which was shown to be downregulated in *APOE4* cases and upregulated in plasma samples of *APOE3* cases in this study. *A1F1* was originally cloned from a rat heart allograft under chronic rejection, and it is involved in several inflammatory conditions including atherosclerosis. Crossbreeding experiments *A1F1* and *APOE* transgenic mice have shown an interaction between these genes leading to atherosclerotic vasculopathy though modulation of the incorporation of degenerated LDL by macrophages [47, 48].

In brain, the resident macrophages, microglia cells, are the specialized phagocytic cells acting through a complement dependent mechanism coupled to ATP production. The analysis of single cell cortex data points to a pivotal role of the glial lineage in the development of AD in accordance with RRA results and current knowledge. Beyond astrocytes and microglia, the main cell types in which *APOE* is expressed, oligodendrocytes and oligodendrocyte precursors (OPCs) also play a role; interestingly, it has been suggested that astrocytes and oligodendrocytes could also participate in phagocytosis in the brain [49]. But the main role of oligodendrocytes is the production of myelin in the central nervous system, a cholesterol dependent mechanism; oligodendrocytes are continuously generated in the healthy adult brain, being the formation of new myelinating oligodendrocytes during adult life an important mechanism for neuroplasticity [50]. Astrocytes were shown to facilitate all steps of myelination, promoting OPC proliferation through *PDGF* and *FGF2*, or inhibiting the differentiation of OPCs into myelin-forming cells through the *CD44* receptor. Furthermore, *CD44* is a top candidate from cortex RRA analysis upregulated in astrocytic cells of AD cases of all *APOE* strata, particularly in APOE4, while downregulated in the other cell types including OPCs, illustrating the complexity of AD related mechanism at the cellular level. Myeloid basic protein encoding gene (*MBP*) is one of the top RRA candidates from the *APOE2* stratum, also reinforcing the relevance of myelination in AD in agreement with recent research in the field [51, 52]. In fact, evidence from multiple sclerosis- lesions suggests that Fc receptors and complement have relevant roles in myelin phagocytosis, while *in-vitro* blockade of Fc or CRs reduced myelin phagocytosis [53].

In summary, through the integration of multi-OMICS datasets we have identified both common and *APOE* specific signatures of AD. The ADAPTED consortium has generated isogenic hiPSC derived macrophages, neurons, astrocytes, and microglia carrying the different APOE haplotypes to further explore presented findings in human samples, in a cell-type specific manner. This will support the further elucidation of APOE dependent pathways that drive the AD risk and potentially support developing a therapy for AD patients.

## MATERIALS AND METHODS

**Figure 7:**
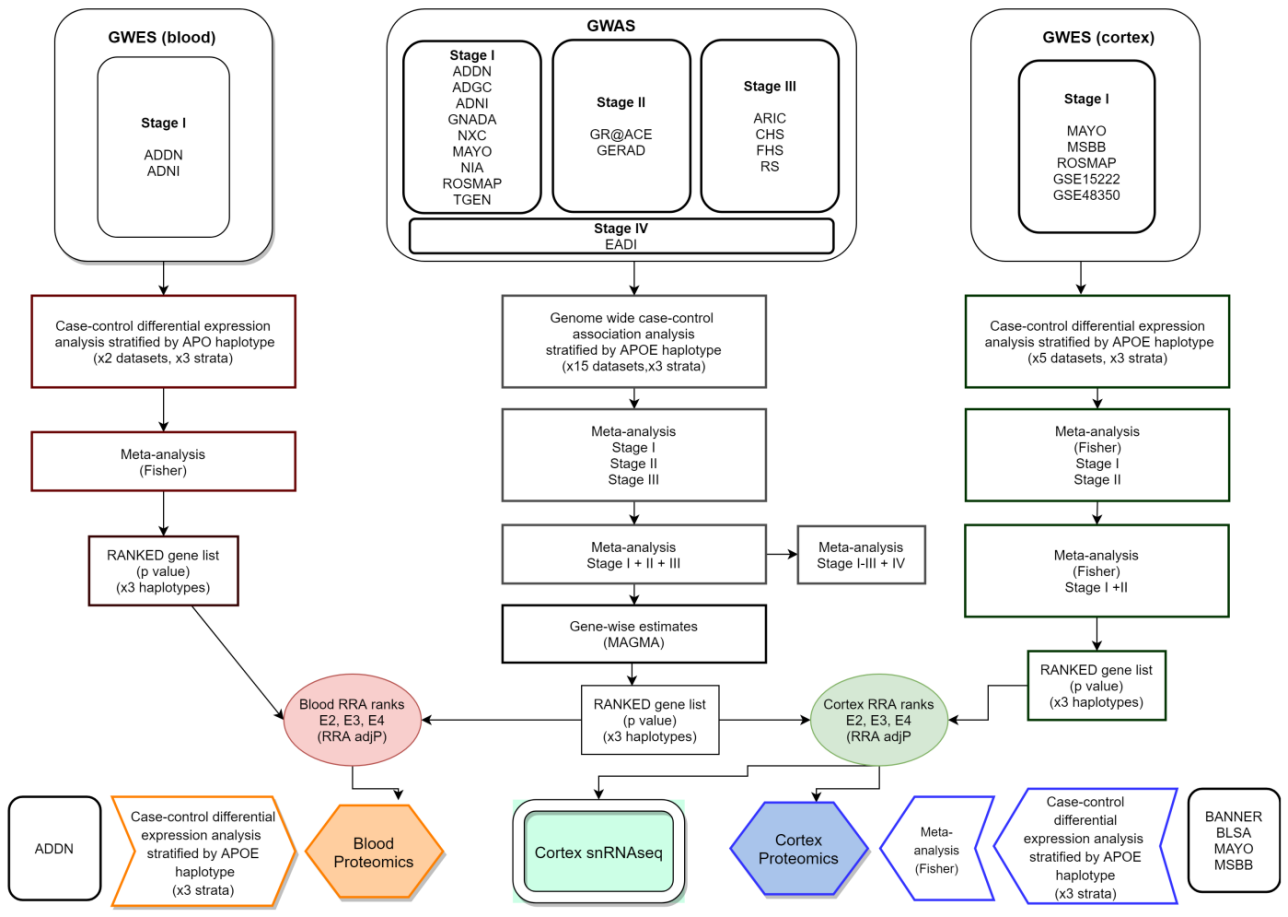
**Integrative analysis workflow.**

Table 2 summarizes the datasets and number of individuals by APOE stratum included at each analysis stage (total number of processed samples: 50,737). A flow chart of the analyses performed in this report is shown in Figure 7. Additional information about study datasets is provided as Supplementary Note.

**Table 2:** Study datasets.

	**ApoE2**			**ApoE3**			**ApoE4**	
	**Controls**	**Cases**		**Controls**	**Cases**		**Controls**	**Cases**
***GWAS Stage I***								
**ADGC**	191	113		810	1070		329	2063
**ADDN**	22	10		126	104		50	140
**ADNI**	40	10		161	153		67	303
**GNADA**	92	35		487	252		164	467
**NXC**	78	21		510	150		128	150
**MAYO**	147	24		657	233		286	478
**NIA**	68	9		353	94		374	297
**ROSMAP**	51	62		153	353		23	200
**TGEN**	71	25		281	261		89	420
***Total Stage I***	***760***	***309***		***3538***	***2670***		***1510***	***4518***
***GWAS Stage II***								
**FACE**	330	123		2220	1314		639	1115
**GERAD**	780	145		3572	1090		1574	1634
***Total Stage II***	***1110***	***268***		***5792***	***2404***		***2213***	***2749***
***GWAS Stage III***								
**ARIC**	1001	144		4479	688		1666	567
**CHS**	243	44		1058	240		302	133
**FHS**	474	27		2346	180		687	88
**RS**	667	102		2770	548		1001	435
***Total Stage III***	***2385***	***317***		***10653***	***1656***		***3656***	***1223***
***GWAS Stage IV***								
**EADI**	819	124		4317	993		1188	1135
***Total Stage IV***	***819***	***124***		***4317***	***993***		***1188***	***1135***
***TOTAL GWAS***	***5074***	***1018***		***24300***	***7723***		***8567***	***9625***
***Blood GWES***								
**ADDN**	14	11		92	79		37	107
**ADNI**	27	3		118	71		48	127
***TOTAL Blood GWES***	***41***	***14***		***210***	***150***		***85***	***234***
***Cortex GWES***								
**MAYO (TCX)**	27	5		112	71		43	118
**ROSMAP (DLPFC)**	28	26		85	147		13	92
**MSBB FP**	2	1		10	16		3	18
**MSBB OVC**	1	3		8	16		4	7
**MSBB DLPF**	2	0		9	12		5	16
**MSBB PCG**	0	3		5	14		0	14
**MSBB PFC**	1	1		9	15		1	17
**GSE15222**	26	3		114	48		37	108
**GSE48350 SFG**	9	1		23	8		16	10
**GSE48350 EC**	8	1		16	5		15	8
**GSE48350 PG**	10	1		21	8		13	14
***TOTAL Cortex GWES***	***114***	***45***		***412***	***360***		***150***	***422***
***Blood Proteomics***								
**ADDN**	12	10		46	76		28	110
***TOTAL Blood Proteomics***	***12***	***10***		***46***	***76***		***28***	***110***
***Brain proteomics***								
**BANNER**	6	6		29	35		6	57
**BLSA**	5	3		7	10		1	7
**MAYO**	3	4		23	37		4	43
**MSBB**	6	10		15	88		7	44
***TOTAL Cortex Proteomics***	***20***	***23***		***74***	***170***		***18***	***151***
***Brain snRNAseq***								
ROSMAP	4	2		7	7		2	9
***Total snRNAseq***	***4***	***2***		***7***	***7***		***2***	***9***
***TOTAL***	***5265***	***1112***		***25049***	***8486***		***8850***	***10551***

### GWAS data

### *Study cohorts*

Stage I GWAS meta-analysis comprised 13,305 subjects from nine datasets, including the Alzheimer's Disease Neuroimaging Initiative (ADNI), the AddNeuroMed study, the Alzheimer’s Disease Genetics Consortium (ADGC), the Multi-Site Collaborative Study for Genotype-Phenotype Associations in Alzheimer’s Disease (GenADA), the Mayo Clinic Alzheimer's Disease Genetic Study, the Neocodex-Murcia study, the National Institute on Aging (NIA) - Late Onset Alzheimer's Disease Family Study, the Religious Orders Study and the Rush Memory and Aging Project (ROSMAP) study and the TGEN study. Stage II meta-analysis (N=14,536 individuals) included the Genome Research at Fundació ACE (GR@ACE) study stage I and the Genetic and Environmental Risk in Alzheimer’s Disease (GERAD) study. The Cohorts for Heart and Ageing Research in Genomic Epidemiology (CHARGE) consortium contributed the Atherosclerosis Risk in Communities (ARIC) study, Cardiovascular Heart Study (CHS), the Framingham study (FS) and the Rotterdam study (RS) for Stage III GWAS meta-analysis (N=11,345 subjects). Additional validation (stage IV) of stage I-III meta-analysis was performed on the European Alzheimer’s Disease Initiative (EADI) dataset (N=8,576 samples). Estimated power for the stage I+II+III meta-analysis was 65.9%, 99.9% and 99.8% for a SNP with MAF=0.2 and OR=1.2 in the E2, E3 and E4 strata respectively, dropping to 28.3%,86.5% and 76.4% for a variant showing MAF=0.2 and OR=1.1.

Whenever possible, clinical information was reviewed to exclude: i) cases not classified as confirmed or probable AD ii) controls with amyloid pathology or history of altered cognition tests.

### *Quality control (QC) and imputation*

A standard QC was applied to all datasets, including removal of individuals with more than 3% missing genotypes, with excess autosomal heterozygosity (>0.35 or more than 3 standard deviations (SD) from population mean), those showing a discrepancy between genotypic and reported sex, as well as individuals of non-European ancestry based on SMARTPCA principal component (PC) analyses (exclusion of subjects more than 6 SDs away from the population mean) [54]. Duplicated and related individuals were identified and removed by means of IBS estimates (IBS>0.1875) both within and across studies. At the genotype level, we removed SNPs with missing genotype rate > 5%, not in Hardy-Weinberg equilibrium (HWE) (p<10^-6^ in controls) and SNPs with minor allele frequency (MAF) < 1%. When necessary, datasets were updated to genome build GRCh37/hg19.

Genotype imputation was performed at the University of Michigan server using the minimac3 algorithm and the SHAPEIT tool for haplotype phasing with the Haplotype Reference Consortium (HRC) cohort as reference panel [55]. After imputation, only SNPs with an R^2^ quality estimate higher than 0.3 and MAF >1% were kept for association analysis.

### *APOE stratified association analysis*

Association analysis was performed within each dataset in three independent groups: ε2 stratum (including subjects with *APOE* genotypes ε2/ε2 and ε2/ε3), ε3 stratum (ε3/ε3 individuals) and ε4 stratum (ε3/ε4 and ε4/ ε4 carriers). The ε2/ε4 genotype was excluded because of the combination of both the protective and deleterious alleles. Association of genotype dosages with the AD case-control status was explored through regression models adjusted by age, sex and the first four PC vectors as covariates using PLINK software [56].

### *Sex and APOE stratified association analysis*

We also explored the effect of both *APOE* and sex on susceptibility to AD using two approaches. We first performed a sex stratified analysis using logistic regression models adjusted by age, the first four PC vectors and *APOE* genotype as a quantitative trait, assigning each allele E2 a value of -1, each E3 allele a value of 0 and each E4 allele a value of +1 (full range: from -2 to 2). Additionally, we performed an association analysis stratified by both *APOE* and sex. For these analyses, eight datasets from Stage I (ADDN, ADGC, ADNI, GNADA, MAYO, NIA, NXC, ROSMAP, N=12,158 individuals) and Stage II (GR@ACE, 5,741 subjects) were used.

### *Meta-analysis (meta-GWAS)*

Within each stage and stratum, association results were combined by meta-analysis using the inverse variance method implemented in METAL [57] or PLINK software programs. SNPs with MAF >1% that were available in at least 60% of the datasets at each stage were included in the meta-analysis. Genomic inflation lambda (*λ*) was calculated using the GenABEL package [58]. Manhattans and QQ plots were generated with the qqman R package [59].

### *Gene-level analysis*

Gene level analysis was performed using MAGMA software, which compute gene-wise statistics taking into account physical distance and linkage disequilibrium (LD) between markers [60]. All SNPs with MAF above 5% were used in these analyses, setting a distance threshold of 50kb. At each stratum, genes were ranked according to the global p mean value.

### Genome-wide expression analysis (GWES) and meta-analysis

### *Study cohorts*

Whole blood expression profiles for meta-analysis were obtained from ADNI and AddNeuroMed studies (N=734). The cortex gene-expression meta-analysis included Mount Sinai Brain Bank (MSBB) dataset (frontal pole, occipital visual cortex, dorsolateral prefrontal cortex, precentral gyrus, prefrontal cortex), ROSMAP (dorsolateral prefrontal cortex) and MAYO (temporal cortex) studies and GSE15222 [61] and GSE48350 [62] (entorhinal cortex, superior frontal cortex, post-central gyrus) datasets from the GEO repository (N=1,503).

### *QC*

For these analyses, we used background corrected and normalized intensity values from expression microarrays distributed by the dataset providers, except for GSE48350. For this GEO dataset, raw. CEL files we downloaded and processed using the Robust Multi-array Average (RMA) algorithm integrated in the affy R package for background correction and normalization [63]. Diagnostic plots included Residuals vs Fitted, Residual vs Leverage, Scale Location, PCA and QQ plots; outlier values identified in these analyses were disregarded. For those datasets provided in different experimental batches, the ComBat function from the sva R package [64] was used to minimize batch effects. A multivariate regression model was fitted to adjust intensity values for covariates, including pH, post-mortem interval (PMI), RNA integrity numbers (RIN), age of death, sex, race and use of lipid lowering medication when available.

### *Differential expression analysis*

As for GWAS data, differential expression (DE) analysis between cases and controls was performed independently in the three *APOE* subgroups using R package limma [65] by dataset and brain region when available. Limma results were adjusted for multiple testing using the Benjamin and Hochberg’s (BH) method. Volcano plots and heatmaps were produced to assess these results. Probes were annotated to gene symbols using appropriate specific libraries, keeping the most differentially expressed mRNA isoform for those genes showing alternative splicing.

### *Differential expression meta-analysis (meta-GWES)*

Independent *APOE* stratified meta-analyses were performed for combining DE results from the different datasets into single ranked gene lists for both blood and cortex. For cortex, only genes present in at least a 70% of the datasets were considered for meta-analysis. Individual logFCs were combined using the Random Effect Model (REM). Given that the analysis included data from different brain regions, genes were ranked according to the Fisher statistics to avoid making assumptions about the directionality of the effect, aimed at identifying candidate markers differentially expressed in the “majority” of studies, where Fisher methods has been described to outperform other methods in terms of power detection, biological association, stability and robustness [66]. All the analyses were performed with the metaDE R tool. Heatmap graphs were generated with the Pheatmap R package.

### Integrative analysis

In order to obtain per-gene single estimates GWAS and GWES data were combined using the Robust Rank Aggregation (RRA) method [67]. The algorithm, integrated in the RobustRankAggreg R package, uses a probabilistic model for aggregation that is robust to noise and also facilitates the calculation of significance probabilities for all the elements in the final ranking. Two independent runs of the RRA algorithm were performed. In all of them we combined stage I+II+III GWAS meta-analysis plus blood or cortex GWES metanalyses (Figure 7). Final gene ranks for blood and cortex were generated according to ascending order of the exact p values generated by the RRA algorithm.

### Proteomic data analysis

Proteomic data from blood (ADDN study) and brain (BANNER, BLSA, MAYO and MSBB studies) were collected. Histograms and boxplots were generated to assess the distribution of normalized intensity protein expression values distributed by data providers. Differential protein expression analyses by study and *APOE* stratum were performed using *limma*, with PMI, age, sex and, when available, lipid lowering medication as covariates. Meta-analysis of the diverse brain datasets was performed as described for GWES datasets.

### Single nuclei RNAseq (snRNAseq) data analysis

Additionally, we explored snRNAseq cortex data from the ROSMAP study [19]. Count matrix provided by ROSMAP study was processed using Seurat package [68]. After QC (filtering out cells that have unique feature counts over 2,500 or less than 200 and cells with >5% mitochondrial counts), data were normalized and scaled. Prior to clustering the cells, we applied the Uniform Manifold Approximation and Projection (UMAP) dimensional reduction technique. Finally, a differential expression analysis between AD cases and controls was performed by each cell type using the edgeR package [69].

### Enrichment analysis

Enrichment analysis of RRA results was performed using four different tools: WebGestaltR [70, 71], FUMA [72] and gPROFILER [73], for genes passing the multiple testing correction threshold (p=0.05), and GSEA [74] for full gene ranked lists. The databases being interrogated include GO, KEGG, WikiPathways, and Reactome. Only pathways and GO categories selected by at least two enrichment tools with adjusted p<0.05 and a minimum of three overlapping genes were selected for further exploring.

### Data availability statement

Summary statistics are included as Supplementary Tables and will be made available through Synapse repository (https://www.synapse.org/) upon publication.

Most data used in this article are publicly available (see acknowledgement section).

### Code availability statement

Code used for this article will be made publicly available through a public Jupyter server (Madrid L, Rubio-Escudero C, Pontes B, González-Pérez A, Riquelme JA, Sáez ME. MOMIC: A Multi-Omics Pipeline for data analysis, integration and interpretation (manuscript in preparation))

## Supplementary Material

Extended datasets description

Supplementary Figures

Supplementary Table 1

Supplementary Table 2

Supplementary Table 3

Supplementary Table 4

Supplementary Table 5

Supplementary Table 6

Supplementary Table 7

Supplementary Table 8

Supplementary Table 9

Supplementary Table 10

Supplementary Table 11

Supplementary Table 12

Supplementary Table 13

Supplementary Table 14

Supplementary Table 15

Supplementary Table 16

Supplementary Table 17

Supplementary Table 18

Supplementary Table 19

Supplementary Table 20

Supplementary Table 21

Supplementary Table 22

Supplementary Table 23

Supplementary Table 24

Supplementary Table 25

Supplementary Table 26

Supplementary Table 27

Supplementary Table 28

Supplementary Table 29

Supplementary Table 30

Supplementary Table 31

Supplementary Table 32

Supplementary Table 33

Supplementary Table 34

Supplementary Table 35

Supplementary Table 36

Supplementary Table 37

Supplementary Table 38

Supplementary Table 39
