# Supplementary material for: Multiomics integrative analysis identifies APOE allele-specific blood biomarkers associated to Alzheimer’s disease etiopathogenesis
Source: Aging (Albany NY). 2021 Apr 12;13(7):9277–329. doi: 10.18632/aging.202950 (PMC8064208; doi:10.18632/aging.202950)
Supplement: Extended datasets description [file aging-13-202950-s001.docx]

EXTENDED DATASETS DESCRIPTION

The Alzheimer's disease genetics consortium (ADGC)

The National Institute on Aging (NIA) Alzheimer's Disease Centres (ADCs) cohort includes subjects ascertained and evaluated by the clinical and neuropathology cores of the 29 NIA-funded ADCs [1]. Data collection was coordinated by the National Alzheimer's Coordinating Center (NACC). The ADC cohort consists of autopsy-confirmed and clinically-confirmed AD cases, and cognitively normal elders (CNEs) with complete neuropathology data who were older than 60 years at age of death, and living CNEs evaluated using the Uniform dataset (UDS) protocol who were documented to not have mild cognitive impairment (MCI) and were between 60 and 100 years of age at assessment.

The AddNeuroMed study

AddNeuroMed was a public-private partnership for biomarker discovery and replication in Alzheimer’s disease [2, 3]. It was designed as a multi-center study in Europe with the first patient enrolled in January 2006 and the last in February 2008. The study protocol was planned for a baseline assessment visit with follow ups every 3 months for the first year, proceeded by annual visits that continued through 2013. The study enrolled a total of 258 AD, 257 MCI and 266 controls, not all with complete data at each assessment.

The Alzheimer’s disease neuroimaging initiative (ADNI)

Data used in the preparation of this article were obtained from the Alzheimer’s Disease Neuroimaging Initiative (ADNI) database (adni.loni.usc.edu). The ADNI was launched in 2003 as a public-private partnership, led by Principal Investigator Michael W. Weiner, MD. The primary goal of ADNI has been to test whether serial magnetic resonance imaging (MRI), positron emission tomography (PET), other biological markers, and clinical and neuropsychological assessment can be combined to measure the progression of mild cognitive impairment (MCI) and early Alzheimer’s disease (AD). The ADNI study has three phases: ADNI1, ADNI GO and ADNI2. For up-to-date information, see [www.adni-info.org](http://www.adni-info.org).

The atherosclerosis risk in communities (ARIC)

The ARIC study is a population-based cohort study of atherosclerosis and clinical atherosclerotic diseases (ARIC Investigators 1989) [4]. At its inception (1987-1989), 15,792 men and women, including 11,478 white and 4,266 black participants were recruited from four U.S. communities: Suburban Minneapolis, Minnesota; Washington County, Maryland; Forsyth County, North Carolina; and Jackson, Mississippi. In the first 3 communities, the sample reflects the demographic composition of the community. In Jackson, only black residents were enrolled. Participants were between age 45 and 64 years at their baseline examination in 1987-1989 when blood was drawn for DNA extraction and participants consented to genetic testing. Vascular risk factors and outcomes, including transient ischemic attack, stroke and dementia, were determined in a standard fashion. During the first 2 years (1993-1994) of the third ARIC examination (V3), participants aged 55 and older from the Forsyth County and Jackson sites were invited to undergo cranial MRI. This subgroup of individuals with MRI scanning represents a random sample of the full cohort because examination dates were allocated at baseline through randomly selected induction cycles.

The Banner Sun Health Research Institute (Banner) study

This study is based on 201 post-mortem brain tissue samples obtained from the Banner Sun Health Research Institute's Brain and Body Donation Program. The tissue set came from 101 cognitively normal (controls) and 100 Alzheimer’s disease (AD) cases. Label free proteome analysis was done on the dorsolateral prefrontal cortex from all individuals. Post-mortem neuropathological evaluation was performed at Banner Sun Health Research Institute. This included amyloid plaque distribution according to CERAD criteria and neurofibrillary tangle pathology assessed with Braak staging. Control cases were defined as cognitively normal within on average 9 months of death with low CERAD (0.13 ±0.35) and Braak (2.26 ±0.94) measures for amyloid and tau neuropathology, respectively. In contrast, AD cases were demented at the last clinical research assessment, and the brains showed high CERAD (2.9 ±0.31) and Braak (5.4 ±0.82) scores consistent with moderate to severe neuropathological burden. There was no significant difference in age or post mortem interval (PMI) between control and AD.

The Baltimore longitudinal study on aging (BLSA) study

We BLSA study included 97 post-mortem brain tissue samples from the National Institute on Aging’s Baltimore Longitudinal Study of Aging (BLSA, <https://www.blsa.nih.gov/>). The tissue set came from 50 individuals representing 15 controls, 15 AsymAD and 20 AD cases. For 47 cases, we analyzed tissue from both the dorsolateral prefrontal cortex (FC, Brodmann Area 9) and precuneus (PC, Brodmann Area 7). Both regions are affected in AD, and PC is a site of early amyloid deposition and glucose hypometabolism. Post-mortem neuropathological evaluation was performed at the Johns Hopkins Alzheimer’s Disease Research Center with the Uniform Data Set including amyloid plaque distribution according to CERAD criteria and neurofibrillary tangle pathology assessed with Braak staging. Control cases were defined as cognitively normal within on average 9 months of death with low CERAD (0.13 ±0.35) and Braak (2.26 ±0.94) measures for amyloid and tau neuropathology, respectively [5]. In contrast, AD cases were demented at the last clinical research assessment, and the brains showed high CERAD (2.9 ±0.31) and Braak (5.4 ±0.82) scores consistent with moderate to severe neuropathological burden. AsymAD cases were cognitively normal proximate to death and had high CERAD (2.1 ±0.52) and moderate Braak (3.6 ±0.99).

The cohort for heart and ageing research in genomic epidemiology (CHARGE) consortium

The CHARGE consortium currently includes six large, prospective, community-based cohort studies that have genome-wide variation data coupled with extensive data on multiple phenotypes [5]. A neurology working-group arrived at a consensus on phenotype harmonization, covariate selection and analytic plans for within-study analyses and meta-analysis of results [6]. Consent procedures, examination and surveillance components, data security, genotyping protocols and study design at each study were approved by a local Institutional Review Board, details are provided below. Of the six studies, we included in this study the Atherosclerosis Risk in Communities (ARIC) study, the Cardiovascular Health Study (CHS), the Framingham Heart Study (FHS) and the Rotterdam Study (RS).

The cardiovascular health study (CHS)

The CHS is a population-based cohort study of risk factors for coronary heart disease and stroke in adults ≥ 65 years conducted across four field centers [7]. The original predominantly European ancestry cohort of 5,201 persons was recruited in 1989-1990 from random samples of the Medicare eligibility lists; subsequently, an additional predominantly African-American cohort of 687 persons was enrolled for a total sample of 5,888. Blood samples were drawn from all participants at their baseline examination and DNA was subsequently extracted from available samples. Genotyping was performed at the General Clinical Research Center’s Phenotyping/Genotyping Laboratory at Cedars-Sinai among CHS participants who consented to genetic testing and had DNA. European ancestry participants were excluded from the GWAS study sample due to the presence at study baseline of coronary heart disease, congestive heart failure, peripheral vascular disease, valvular heart disease, stroke or transient ischemic attack or lack of available DNA. Among those with successful GWAS, 567 European ancestry participants had available FreeSurfer measures for this analysis. CHS was approved by institutional review committees at each field center and individuals in the present analysis had available DNA and gave informed consent including consent to use of genetic information for the study of cardiovascular disease.

The European Alzheimer’s disease initiative (EADI) consortium

All the 2,240 Alzheimer’s disease cases were ascertained by neurologists from Bordeaux, Dijon, Lille, Montpellier, Paris, Rouen, and were identified as French NHW ancestry. Clinical diagnosis of probable Alzheimer’s disease was established according to the DSM-III-R and NINCDS-ADRDA criteria. Controls were selected from the 3C Study [8]. This cohort is a population-based, prospective (10-years follow-up) study of the relationship between vascular factors and dementia. It has been carried out in three French cities: Bordeaux (southwest France), Montpellier (southeast France) and Dijon (central eastern France). A sample of non-institutionalized, over-65 subjects was randomly selected from the electoral rolls of each city. Between January 1999 and March 2001, 9,686 subjects meeting the inclusion criteria agreed to participate. Following recruitment, 392 subjects withdrew from the study. Thus, 9,294 subjects were finally included in the study (2,104 in Bordeaux, 4,931 in Dijon and 2,259 in Montpellier). Genomic DNA samples of 7,200 individuals were transferred to the French Centre National de Génotypage (CNG). First stage samples that passed DNA quality control were genotyped with Illumina Human 610-Quad BeadChips. At the end we removed 308 samples because they were found to be first- or second-degree relatives of other study participants or were assessed non-European descent based on genetic analysis using methods described in 89. In this final sample,
at 10 years of follow-up, 564 individuals suffered
from Alzheimer’s disease with 95 prevalent and 469 incident cases.

The Framingham heart study (FHS)

The FHS is a three-generation, single-site, community-based, ongoing cohort study that was initiated in 1948 to investigate the risk factors for cardiovascular disease. It now comprises 3 generations of participants: the Original cohort followed since 19489; their Offspring and spouses of the Offspring (Gen 2), followed since 1971 [9]; and children from the largest Offspring families enrolled in 2000 (Gen 3) [10]. The Original cohort enrolled 5,209 men and women who comprised two-thirds of the adult population then residing in Framingham, MA. Survivors continue to receive biennial examinations. The Offspring cohort comprises 5,124 persons (including 3,514 biological offspring) who have been examined approximately once every 4 years. The Third-generation includes 4,095 participants with at least one parent in the Offspring Cohort. The first two generations were invited to undergo an initial brain MRI in 1999-2005, and for Gen 3, brain MRI began in 2009. The population of Framingham was virtually entirely white (Europeans of English, Scots, Irish and Italian descent) in 1948 when the Original cohort was recruited. Self-reports of ethnicity across all three generations were 99.7% whites, reflecting the ethnicity of the population of Framingham in 1948. FHS participants had DNA extracted and provided consent for genotyping, and eligible participants underwent genome-wide genotyping.

Multi-site collaborative study for genotype-phenotype associations in Alzheimer's disease and longitudinal follow-up of genotype-phenotype associations in Alzheimer's disease and neuroimaging component of genotype-phenotype associations in Alzheimer's disease (GenADA)

GenADA was a multi-site collaborative study, involving GlaxoSmithKline Inc and nine medical centers in Canada, including 1000 AD patients and 1000 ethnically-matched controls in order to associate DNA sequence (allelic) variations in candidate genes with AD phenotypes [11, 12]. The study consists of both retrospective and prospective data. Where possible, biological relatives with Alzheimer's (up to third degree relationship) and unaffected siblings of AD cases were also recruited.

The genetic and environmental risk for Alzheimer’s disease (GERAD1) consortium

The GERAD1 sample comprised up to 3941 AD cases and 7848 controls. A subset of this sample has been used in this study and were genotyped at the Sanger Institute on the Illumina 610-quad chip. These samples were recruited by the Medical Research Council (MRC) Genetic Resource for AD (Cardiff University; Kings College London; Cambridge University; Trinity College Dublin), the Alzheimer’s Research UK (ARUK) Collaboration (University of Nottingham; University of Manchester; University of Southampton; University of Bristol; Queen’s University Belfast; the Oxford Project to Investigate Memory and Ageing (OPTIMA), Oxford University); Washington University, St Louis, United States; MRC PRION Unit, University College London; London and the South East Region AD project (LASER-AD), University College London; Competence Network of Dementia (CND) and Department of Psychiatry, University of Bonn, Germany and the National Institute of Mental Health (NIMH) AD Genetics Initiative. All AD cases met criteria for either probable (NINCDS-ADRDA, DSM-IV) or definite (CERAD) AD. All elderly controls were screened for dementia using the MMSE or ADAS-cog, were determined to be free from dementia at neuropathological examination or had a Braak score of 2.5 or lower.”

The genome research @ fundació ACE project (GR@ACE) study

The GR@ACE study comprises 4,120 AD cases and 3,289 control individuals. Cases were recruited from Fundació ACE, Institut Català de Neurociències Aplicades (Catalonia, Spain). Diagnoses were established by a multidisciplinary working-group, including neurologists, neuropsychologists, and social workers, according to the DSM-IV criteria for dementia and to the National Institute on Aging and Alzheimer’s Association’s (NIA-AA) 2011 guidelines for defining AD [13]. Dementia individuals diagnosed with probable or possible AD at any moment of their clinical course were considered AD cases.

Briefly, participants were genotyped using the Axiom 815K Spanish Biobank Array (Thermo Fisher), performed in the Spanish National Center for Genotyping (CeGEN, Santiago de Compostela, Spain). Individuals were excluded for low-quality samples, (call rate <97%), excess heterozygosity, sample duplicates, or relation to another sample (PIHAT > 0.1875). Individuals were excluded if sex discrepancy was detected. Population outliers of European ancestry were also removed. Variants were excluded if they departed from the Hardy-Weinberg equilibrium (P-value ≤ 1 × 10-6), presented a different missing rate between cases and controls (P-value < 5 × 10-4 for the difference), or had a low frequency (MAF < 0.01) or low call rate < 95%. High-quality variants
were imputed in Michigan Server using the
Haplotype reference consortium (HRC) panel ([https://
imputationserver.sph.umich.edu](https://imputationserver.sph.umich.edu)). Only high imputation quality markers (MAF > 0.05 and R2>0·03) were used for downstream analysis. Further information about phenotyping and GWAS quality controls have been previously provided [14].

The Mayo clinic LOAD genome-wide association study (MAYO)

Subjects from the Mayo LOAD GWAS were selected from two clinical AD Case-Control series: Mayo Clinic Jacksonville (MCJ), Mayo Clinic Rochester (MCR)and a neuropathological series of autopsy-confirmed subjects from the Mayo Clinic Brain Bank [15]. All subjects from the clinical series (MCJ and MCR) were diagnosed by a Mayo Clinic neurologist; all control subjects had a Clinical Dementia Rating score of zero at the most recent time of testing; all LOAD patients had a diagnosis of probable or possible AD according to the NINCDS-ADRDA criteria [16]. All ADs had definite diagnosis according to the NINCDS-ADRDA criteria and had Braak scores of ≥4.0. All non–AD Controls had Braak scores of ≤2.5; many had brain pathology unrelated to AD.

The Mount Sinai brain bank (MSBB) study

Brain specimens were obtained from the Mount Sinai/JJ Peters VA Medical Center Brain Bank (MSBB) which holds over 1,700 samples. This cohort was assembled after applying stringent inclusion/exclusion criteria and represents the full spectrum of disease severity. Neuropathological assessments are performed according to the Consortium to Establish a Registry for Alzheimer's Disease (CERAD) protocol and include assessment by hematoxylin and eosin, modified Bielschowski, modified thioflavin S, and anti-β amyloid (4G8), anti-tau (AD2) and anti-ubiquitin (Daka Corp.). Each case is assigned a Braak AD-staging score for progression of neurofibrillary neuropathology. Quantitative data regarding the density of neuritic plaques in the middle frontal gyrus, orbital frontal cortex, superior temporal gyrus, inferior parietal cortex and calcarine cortex are also collected as described. Clinical dementia rating scale (CDR) and mini–mental state examination (MMSE) severity tests are conducted for assessment of dementia and cognitive status. Final diagnoses and CDR scores are conferred by consensus. Based on CDR classification, subjects are grouped as no cognitive deficits (CDR = 0), questionable dementia (CDR = 0.5), mild dementia (CDR = 1.0), moderate dementia (CDR = 2.0), and severe to terminal dementia (CDR = 3.0–5.0). Covariates including demographic and neuropathological data were collected on the samples used for this project including postmortem interval, race, age of death, clinical dementia rating, clinical neuropathology diagnosis, CERAD, Braak, sex, and a series of neuropathological variables.

The Neocodex-Murcia study (NXC)

The study includes 327 sporadic AD patients and 801 controls with unknown cognitive status from the Spanish general population collected by
Neocodex [17, 18]. AD patients were diagnosed as possible or probable AD in accordance with the
criteria of the National Institute of Neurological and Communicative Disorders and Stroke and the Alzheimer's Disease and Related Disorders Association (NINCDS-ADRDA) [16].

The National Institute on Aging - late onset Alzheimer's disease family study (NIA)

The goal of this study is to identify and recruit families with two or more siblings with the late-onset form of Alzheimer's disease and a cohort of unrelated, non-demented controls similar in age and ethnic background, and to make the samples, the clinical and genotyping data and preliminary analyses available to qualified investigators world-wide [19]. Genotyping by the Center for Inherited Disease Research (CIDR) was performed using the Illumina Infinium II assay protocol with hybridization to Illumina Human 610Quadv1_B Beadchips.

The religious orders study and memory and aging project (ROS/MAP) study

The Religious Orders Study (ROS) is a longitudinal clinical-pathologic cohort study of aging and Alzheimer's disease (AD) from the Rush University that enrolled individuals from religious communities for longitudinal clinical analysis and brain donation [20]. Participants were enrolled from more than 40 groups of religious orders (nuns, priests, brothers) across the United States. Medical conditions are documented starting in 1994 by clinical evaluation or self-report. Alzheimer's Disease status was determined by a computer algorithm based on cognitive test performance with a series of discrete clinical judgments made in series by a neuropsychologist and a clinician.

The Memory and Aging Project (MAP) is a longitudinal, epidemiologic clinical-pathologic cohort study of common chronic conditions of aging with an emphasis on decline in cognitive and motor function and risk of Alzheimer’s disease that began in 1997 and is run from Rush University [20]. This study was designed to complement the ROS study by enrolling individuals with a wider range of life experiences and socioeconomic status into a study of similar structure and design as ROS. The study enrolled older individuals without any signs of dementia, primarily recruiting from continuous care retirement communities throughout north-eastern Illinois, USA. Diagnoses of dementia
and AD are performed in an identical manner to the ROS study.

The Rotterdam study

The Rotterdam Study is a prospective, population-based cohort study among individuals living in the well-defined Ommoord district in the city of Rotterdam in The Netherlands [21, 22]. The aim of the study is to determine the occurrence of cardiovascular, neurological, ophthalmic, endocrine, hepatic, respiratory, and psychiatric diseases in elderly people. The cohort was initially defined in 1990 among approximately 7,900 persons, aged 55 years and older, who underwent a home interview and extensive physical examination at the baseline and during follow-up rounds every 3-4 years (RS-I). The cohort was extended in 2000/2001 (RS-II, 3,011 individuals aged 55 years and older) and 2006/2008 (RS-III, 3,932 subjects, aged 45 and older). Written informed consent was obtained from all participants and the Medical Ethics Committee of the Erasmus Medical Center, Rotterdam, approved the study.

The Translational Genomics Research Institute (TGEN) study

The TGEN GWAS study included 643 late onset AD cases and 404 controls from a neuropathological cohort, and 197 late onset AD cases and 114 controls from a clinical cohort [23].

CONSORTIA MEMBERSHIP

ADAPTED consortium

Margot Bakker^1^, Lamiaa Bahnassawy^1^, Peter Reinhardt^1^, Janina Ried^1^, Eric Mohler^1^, Heike Hering^2^, Maria Eugenia Sáez^3^, Antonio González-Pérez3, Laura Madrid^3^,Santos Mañes^4^, Keiryn Bennett^5^, Cornelia van Duijn^6^, Shahzad Ahmad^6^, Agustin Ruiz^7^, Adela Orellana^7^, Pablo García^7^, Itziar de Rojas^7^, Carlos Vicario^8^, Eva Díaz-Guerra^8^, Esther Arribas-González^8^, Alfredo Cabrera^9^, Thomas Hankemeier^10^, Isabelle Kohker^10^, Remko van Vught^11^, Tania Fowke^11^, Bridget Glaysher^12^, Michael Peitz^13^, Alfredo Ramírez^13^, Pamela Martino Adami^13^, Carolina Dalmasso^13^, Luca Kleineidam^13^.

^1^ AbbVie Deutschland GmbH & Co. KG, Genomics Research Center, Knollstrasse, 67061 Ludwigshafen, Germany; ^2^ Biogen Inc., Cambridge, MA, USA; ^3^ Andalusion Bioiformatics Research Centre (CAEBi)Sevilla, Spain; ^4^ Department of Immunology and Oncology, Centro Nacional de Biotecnología (CNB/CSIC); ^5^ DC Biosciences, James Lindsay Place, Dundee, DD1 5JJ, UK; ^6^ Department of Epidemiology, Erasmus MC, Rotterdam, The Netherlands; ^7^ Research Center and Memory clinic Fundació ACE, Institut Català de Neurociències Aplicades, Universitat Internacional de Catalunya, Barcelona, Spain; ^8^ Instituto Cajal , CSIC , Madrid, Spain; ^9^ . Janssen Research and Development, a Division of Janssen Pharmaceutica N.V., Beerse, Belgium; ^10^ Analytical Biosciences, Leiden Academic Centre for Drug Research, Leiden University, Leiden, Netherlands; ^11^ MIMETAS BV, JH Oortweg 19, Leiden, CH, 2333 The Netherlands; ^12^ MODUS Research and Innovation Ltd., Unit D Tayside Software Centre, Gemini Crescent, Dundee Technology Park, Dundee, DD2 1TY, UK; ^13^ Division of Neurogenetics and Molecular Psychiatry, Department of Psychiatry and Psychotherapy, Medical Faculty, University of Cologne, Cologne, Germany.

Cohorts for heart and aging research in genomic epidemiology consortium (CHARGE) consortium

Maria Vronskaya, Aura Frizatti, Nandini Badarinarayan, Rachel Raybould, Taniesha Morgan, Per Hoffmann, Denise Harold, Amy Gerrish, Nicola Denning, Nick C. Fox, Joseph T. Hughes, Yogen Patel, Makrina Daniilidou, James Uphill, Daniela Galimberti, Elio Scarpini, Johannes Kornhuber, Sabrina Sordon, Manuel Mayhaus, Wei Gu, Annette M Hartmann, Simon Lovestone, Rebecca Sussams, Clive Holmes, Wolfgang Maier, Amit Kawalia, Susanne Moebus, James Turton, Jenny Lord, Iwona Kloszewska, Aoibhinn Lynch, Brian Lawlor, Michael Gill, Monica Diez-Fairen, Ignacio Alvarez, Antonio Ciaramella, Chiara Cupidi, Raffaele Giovanni Maletta, Roberta Cecchetti, Magda Tsolaki, David Craig, Despoina Avramidou, Antonia Germanou, Maria Koutroumani, Olymbia Gkatzima, Harald Hampel, David C. Rubinsztein, Lutz Frölich, Bernadette McGuinness, Janet A. Johnston, Peter Passmore, Jonathan M. Schott, Jason D. Warren, Michelle K. Lupton, Petra Proitsi, John Powell, John S. K. Kauwe, Michelangelo Mancuso, Ubaldo Bonuccelli, Andrew McQuillin, Gill Livingston, Nicholas J. Bass, John Hardy, Jose Bras, Minerva M. Carrasquillo, Rita Guerreiro, Elizabeth Fisher, Carlo Masullo, Gina Bisceglio, Li Ma, Neill R. Graff-Radford, Angela Hodges, Martin Scherer, Matthias Riemenschneider, Reinhard Heun, Heike Kölsch, Markus Leber, Isabella Heuser, Ina Giegling, Michael Hüll, John Morris, Kevin Mayo, Thomas Feulner, Dmitriy Drichel, Thomas D. Cushion, Paul Hollingworth, Rachel Marshall, Alun Meggy, Georgina Menzies, Ganna Leonenko, Detelina Grozeva, Giancarlo Russo, Frank Jessen, Bruno Vellas, Emma Vardy, Karl-Heinz Jöckel, Martin Dichgans, David Mann, Stuart Pickering-Brown, Norman Klopp, H-Erich Wichmann, Kevin Morgan, Kristelle Brown, Christopher Medway, Markus M. Nöthen, Nigel M. Hooper, Antonio Daniele, Anthony Bayer, John Gallacher, Hendrik van den Bussche, Carol Brayne, Steffi Riedel-Heller, Ammar Al-Chalabi, Christopher E. Shaw, Jens Wiltfang, Victoria Alvarez, Andrew B. Singleton, John Collinge, Simon Mead, Martin Rossor, Natalie S. Ryan, Benedetta Nacmias, Sandro Sorbi, Eleonora Sacchinelli, Gianfranco Spalletta, Carlo Caltagirone, Maria Donata Orfei, Robert Clarke, A. David Smith, Donald Warden, Gordon Wilcock, Amalia Cecilia Bruni, Maura Gallo, Yoav Ben-Shlomo, Patrizia Mecocci, Pau Pastor, Oliver Peters, Virginia Boccardi, Nick Warner, Panagiotis Deloukas, Carlos Cruchaga, Rhian Gwilliam, Patrick G. Kehoe, Seth Love, Chris Corcoran, JoAnn Tschanz, Ron Munger, Michael C. O’Donovan, Lesley Jones, Michael J. Owen, Valentina Escott-Price, Alfredo Ramirez, Peter A. Holmans, Julie Williams.

The European Alzheimer's disease initiative (EADI) consortium

Benjamin Grenier-Boley, Vincent Damotte, Anne Boland, Céline Bellenguez, Kristel Sleegers, Robert Olaso, Mikko Hiltunen, Jacques Epelbaum, Jean-Guillaume Garnier, Marie-Laure Moutet, Delphine Bacq, Maria Del Zompo, Ignacio Mateo, Florentino Sanchez-Garcia, Maria Candida Deniz Naranjo, David Wallon, Fabienne Garzia, Bertrand Fin, Stéphane Meslage, Sebastiaan Engelborghs, Rik Vandenberghe, Peter De Deyn, Alessio Squassina, Eloy Rodriguez-Rodriguez, Carmen Munoz-Fernadez, Yolanda Aladro Benito, Hakan Thonberg, Vilmantas Giedraitis, Lena Kilander, RoseMarie Brundin, Letizia Concari, Seppo Helisalmi, Anne Maria Koivisto, Annakaisa Haapasalo, Vincenzo Solfrizzi, Vincenza Frisardi, Vincent Deramecourt, Francesca Salani, Nathalie Fievet, Olivier Hanon, Carole Dufouil, Alexis Brice, Karen Ritchie, Bruno Dubois, Hilkka Soininen, Laura Fratiglioni, Lina Keller, Francesco Panza, Didier Hannequin, Paolo Caffarra, Lars Lannfelt, Florence Pasquier, Paola Bossù, Alberto Pilotto, Maria J. Bullido, Paola Bosco, Eliecer Coto, Alberto Lleo, Martin Ingelsson, Caroline Graff, Pascual Sanchez-Juan, Claudine Berr, Stéphanie Debette, Jean-Francois Dartigues, Gael Nicolas, Dominique Campion, Jordi Clarimon, Christine Van Broeckhoven, Jean-François Deleuze, Phillippe Amouyel, Jean-Charles Lambert.

The GERAD study

GERAD1 Collaborators: Denise Harold^1^, Rebecca Sims^1^, Amy Gerrish^1^, Jade Chapman^1^, Valentina Escott-Price^1^, Nandini Badarinarayan^1^, Richard Abraham^1^, Paul Hollingworth^1^, Marian Hamshere^1^, Jaspreet Singh Pahwa^1^, Kimberley Dowzell^1^, Amy Williams^1^, Nicola Jones^1^, Charlene Thomas^1^, Alexandra Stretton^1^, Angharad Morgan^1^, Kate Williams^1^, Sarah Taylor^1^, Simon Lovestone^2^, John Powell^3^, Petroula Proitsi^3^, Michelle K Lupton^3^, Carol Brayne^4^, David C. Rubinsztein^5^, Michael Gill^6^, Brian Lawlor^6^, Aoibhinn Lynch^6^, Kevin Morgan^7^, Kristelle Brown^7^, Peter Passmore^8^, David Craig^8^, Bernadette McGuinness^8^, Janet A Johnston^8^, Stephen Todd^8^, Clive Holmes^9^, David Mann^10^, A. David Smith^11^, Seth Love^12^, Patrick G. Kehoe^12^, John Hardy^13^, Rita Guerreiro^14,15^, Andrew Singleton^14^, Simon Mead^16^, Nick Fox^17^, Martin Rossor^17^, John Collinge^16^, Wolfgang Maier^18^, Frank Jessen^18^, Reiner Heun^18^, Britta Schürmann^18,19^, Alfredo Ramirez^18^, Tim Becker^20^_,_ Christine Herold^20^, André Lacour^20^, Dmitriy Drichel^20^, Hendrik van den Bussche^21^, Isabella Heuser^22^, Johannes Kornhuber^23^, Jens Wiltfang^24^, Martin Dichgans^25,26^, Lutz Frölich^27^, Harald Hampel^28^, Michael Hüll^29^, Dan Rujescu^30^, Alison Goate^31^, John S.K. Kauwe^32^, Carlos Cruchaga^33^, Petra Nowotny^33^, John C. Morris^33^, Kevin Mayo^33^, Gill Livingston^34^, Nicholas J. Bass^34^, Hugh Gurling^34^, Andrew McQuillin^34^, Rhian Gwilliam^35^, Panagiotis Deloukas^35^, Markus M. Nöthen^20^, Peter Holmans^1^, Michael O’Donovan^1^, Michael J.Owen^1^, Julie Williams^1^.

^1^Medical Research Council (MRC) Centre for Neuropsychiatric Genetics and Genomics, Neurosciences and Mental Health Research Institute, Department of Psychological Medicine and Neurology, School of Medicine, Cardiff University, Cardiff, UK. ^2^Department of Psychiatry, Medical Sciences Division, University of Oxford, Oxford, UK. ^3^Kings College London, Institute of Psychiatry, Department of Neuroscience, De Crespigny Park, Denmark Hill, London, UK. ^4^Institute of Public Health, University of Cambridge, Cambridge, UK. ^5^Cambridge Institute for Medical Research, University of Cambridge, Cambridge, UK. ^6^Mercers Institute for Research on Aging, St. James Hospital and Trinity College, Dublin, Ireland. ^7^Institute of Genetics, Queens Medical Centre, University of Nottingham, UK. ^8^Ageing Group, Centre for Public Health, School of Medicine, Dentistry and Biomedical Sciences, Queens University Belfast, UK. ^9^Division of Clinical Neurosciences, School of Medicine, University of Southampton, Southampton, UK. ^10^Clinical Neuroscience Research Group, Greater Manchester Neurosciences Centre, University of Manchester, Salford, UK. ^11^Oxford Project to Investigate Memory and Ageing (OPTIMA), University of Oxford, Department of Pharmacology, Mansfield Road, Oxford, UK. ^12^University of Bristol Institute of Clinical Neurosciences, School of Clinical Sciences, Frenchay Hospital, Bristol, UK. ^13^Department of Molecular Neuroscience and Reta Lilla Weston Laboratories, Institute of Neurology, UCL, London, UK. ^14^Laboratory of Neurogenetics, National Institute on Aging, National Institutes of Health, Bethesda, Maryland, United States of America. ^15^Department of Molecular Neuroscience, Institute of Neurology, University College London, Queen Square, London WC1N 3BG, UK. ^16^MRC Prion Unit, Department of Neurodegenerative Disease, UCL Institute of Neurology, London, UK. ^17^Dementia Research Centre, Department of Neurodegenerative Diseases, University College London, Institute of Neurology, London, UK. ^18^Department of Psychiatry, University of Bonn, Sigmund-Freud-Straβe 25, 53105 Bonn, Germany. ^19^Institute for Molecular Psychiatry, University of Bonn, Bonn, Germany. ^20^Department of Genomics, Life and Brain Center, University of Bonn, Bonn, Germany. ^21^Institute of Primary Medical Care, University Medical Center Hamburg-Eppendorf, Germany. ^22^Department of Psychiatry, Charité Berlin, Germany. ^23^Department of Psychiatry, Friedrich-Alexander-University Erlangen-Nürnberg, Germany. ^24^Department of Psychiatry and Psychotherapy, University Medical Center (UMG), Georg-August-University, Göttingen, Germany. ^25^Institute for Stroke and Dementia Research, Klinikum der Universität München, Marchioninistr. 15, 81377, Munich, Germany. ^26^Department of Neurology, Klinikum der Universität München, Marchioninistr. 15, 81377, Munich, Germany. ^27^Central Institute of Mental Health, Medical Faculty Mannheim, University of Heidelberg, Germany. ^28^Institute for Memory and Alzheimer’s Disease and INSERM, Sorbonne Universities, Pierre and Marie Curie University, Paris, France; Institute for Brain and Spinal Cord Disorders (ICM), Department of Neurology, Hospital of Pitié-Salpétrière, Paris, France. ^29^Centre for Geriatric Medicine and Section of Gerontopsychiatry and Neuropsychology, Medical School, University of Freiburg, Germany. ^30^Department of Psychiatry, University of Halle, Halle, Germany. ^31^Neuroscience Department, Icahn School of Medicine at Mount Sinai, New York, US. ^32^Department of Biology, Brigham Young University, Provo, UT, 84602, USA. ^33^Departments of Psychiatry, Neurology and Genetics, Washington University School of Medicine, St Louis, MO 63110, US. ^34^Department of Mental Health Sciences, University College London, UK. ^35^The Wellcome Trust Sanger Institute, Wellcome Trust Genome Campus, Hinxton, Cambridge, UK.

The GR@ACE/DEGESCO consortium

The GR@ACE study group: Abdelnour C^1,2^, Aguilera N^1^, Alarcon E^1,3^, Alegret M^1,2^, Benaque A^1^, Boada M^1,2^, Buendia M^1^, Cañabate P^1,2^, Carracedo A^4,5^, Corbatón A^6^, de Rojas I^1^, Diego S^1^, Espinosa A^1,2^, Gailhajenet A^1^, García González P^1^, Gil S^1^, Guitart M^1^, González Pérez A^7^, Hernández I^1,2^, Ibarria, M^1^, Lafuente A^1^, Macias J^8^, Maroñas O^4^, Martín E^1^, Martínez MT^6^, Marquié M^1^, Mauleón A^1^, Monté-Rubio G^1^, Montrreal L^1^, Moreno-Grau S^1,2^, Moreno M^1^, Orellana A^1^, Ortega G^1,2^, Pancho A^1^, Pelejà E^1^, Pérez-Cordon A^1^, Pineda JA^8^, Preckler S^1^, Quintela I^3^, Real LM^3,8^, Rodríguez-Gómez O^1,2^, Rosende-Roca M^1^, Ruiz A^1,2^, Ruiz S^1,2^, Sáez ME^7^, Sanabria A^1,2^, Santos-Santos MA^1^, Serrano-Rios M^6^, Sotolongo-Grau O^1^, Tárraga L^1,2^, Valero S^1,2^, Vargas L^1^ (1. Research Center and Memory clinic Fundació ACE. Institut Català de Neurociències Aplicades. Universitat Internacional de Catalunya. Barcelona, Spain; 2. CIBERNED, Center for Networked Biomedical Research on Neurodegenerative Diseases, National Institute of Health Carlos III, Ministry of Economy and Competitiveness, Spain; 3. Dep. of Surgery, Biochemistry and Molecular Biology, School of Medicine. University of Málaga. Málaga, Spain; 4. Grupo de Medicina Xenómica, Centro Nacional de Genotipado (CEGEN-PRB3-ISCIII). Universidade de Santiago de Compostela, Santiago de Compostela, Spain; 5. Fundación Pública Galega de Medicina Xenómica- CIBERER-IDIS, Santiago de Compostela, Spain; 6. Centro de Investigación Biomédica en Red de Diabetes y Enfermedades Metabólicas Asociadas, CIBERDEM, Spain, Hospital Clínico San Carlos, Madrid, Spain; 7. CAEBI. Centro Andaluz de Estudios Bioinformáticos., Sevilla, Spain; 8. Unidad Clínica de Enfermedades Infecciosas y Microbiología. Hospital Universitario de Valme, Sevilla, Spain).

DEGESCO consortium: Adarmes-Gómez AD^1,2^, Alarcón-Martín E^3,4^, Álvarez I^5^, Álvarez V^6,7^, Amer-Ferrer G^8^, Antequera M^9^, Antúnez C^9^, Baquero M^10^, Bernal M^11^, Blesa R^2,12^, Boada M^2,3^, Buiza-Rueda D^1,2^, Bullido MJ^2,13,14^, Burguera JA^10^, Calero M^2,15,16^, Carrillo F^1,2^, Carrión-Claro M^1,2^, Casajeros MJ^17^, Clarimón J^2,12^, Cruz-Gamero JM^4^, de Pancorbo MM^18^, de Rojas I^3^, del Ser T^14^, Diez-Fairen M^5^, Fortea J^2,12^, Franco E^11^, Frank-García A^2,14,19^, García-Alberca JM^20^, Garcia Madrona S^16^, Garcia-Ribas G^16^, Gómez-Garre P^1,2^, Hernández I^2,3^, Hevilla S^20^, Jesús S^1,2^, Labrador Espinosa MA^1,2^, Lage C^2,21^, Legaz A^9^, Lleó A^2,12^, López de Munáin A^22^, López-García S^2,21^, Macias D^1,2^, Manzanares S^9,23^, Marín M^11^, Marín-Muñoz J^9^, Marín T^20^, Marquié M^3^, Martín Montes A^2,13,19^, Martínez B^9^, Martínez C^7,24^, Martínez V^9^, Martínez-Lage Álvarez P^25^, Medina M^2,14^, Mendioroz Iriarte M^26^, Menéndez-González M^7,27^, Mir P^1,2^, Molinuevo JL^28^, Monté-Rubio G^3^, Montrreal L^3^, Moreno-Grau S^2,3^, Orellana A^3^, Pastor AB^15^, Pastor P^5^, Pérez Tur J^2,29,30^, Periñán-Tocino T^1,2^, Piñol Ripoll G^2,31^, Rábano A^2,15,32^, Real de Asúa D^33^, Rodrigo S^11^, Rodríguez-Rodríguez E^2,21^, Royo JL^4^, Ruiz A^2,3^, Sanchez del Valle Díaz R^34^, Sánchez-Juan P^2,21^, Sastre I^2,13^, Sotolongo-Grau O3, Tárraga L^2,3^, Valero S^2,3^, Vicente MP^9^, Vivancos L^9^ (1. Unidad de Trastornos del Movimiento, Servicio de Neurología y Neurofisiología. Instituto de Biomedicina de Sevilla (IBiS), Hospital Universitario Virgen del Rocío/CSIC/Universidad de Sevilla, Seville, Spain; 2. CIBERNED, Network Center for Biomedical Research in Neurodegenerative Diseases, National Institute of Health Carlos III, Spain; 3. Research Center and Memory clinic Fundació ACE. Institut Català de Neurociències Aplicades. Universitat Internacional de Catalunya. Barcelona, Spain; 4. Dep. of Surgery, Biochemistry and Molecular Biology, School of Medicine. University of Málaga. Málaga, Spain; 5. Fundació per la Recerca Biomèdica i Social Mútua Terrassa, and Memory Disorders Unit, Department of Neurology, Hospital Universitari Mutua de Terrassa, University of Barcelona School of Medicine, Terrassa, Barcelona, Spain; 6. Laboratorio de Genética Hospital Universitario Central de Asturias, Oviedo; 7. Instituto de Investigación Biosanitaria del Principado de Asturias (ISPA); 8. Department of Neurology, Hospital Universitario Son Espases, Palma, Spain; 9. Unidad de Demencias. Hospital Clínico Universitario Virgen de la Arrixaca; 10. Servei de Neurologia, Hospital Universitari i Politècnic La Fe; 11. Unidad de Demencias, Servicio de Neurología y Neurofisiología. Instituto de Biomedicina de Sevilla (IBiS), Hospital Universitario Virgen del Rocío/CSIC/Universidad de Sevilla, Seville, Spain; 12. Memory Unit, Neurology Department and Sant Pau Biomedical Research Institute, Hospital de la Santa Creu i Sant Pau, Universitat Autònoma de Barcelona, Barcelona, Spain; 13. Centro de Biologia Molecular Severo Ochoa (C.S.I.C.-U.A.M.), Universidad Autonoma de Madrid, Madrid, Spain; 14. Instituto de Investigacion Sanitaria ‘Hospital la Paz’ (IdIPaz), Madrid, Spain; 15. CIEN Foundation, Queen Sofia Foundation Alzheimer Center, Madrid, Spain; 16. Instituto de Salud Carlos III (ISCIII), Madrid, Spain; 17. Hospital Universitario Ramón y Cajal; Madrid, Spain; 18. BIOMICs, País Vasco; Centro de Investigación Lascaray. Universidad del País Vasco UPV/EHU; 19. Neurology Service, Hospital Universitario La Paz (UAM), Madrid, Spain; 20. Alzheimer Research Center and Memory Clinic. Andalusian Institute for Neuroscience. Málaga, Spain; 21. Neurology Service, Marqués de Valdecilla University Hospital (University of Cantabria and IDIVAL), Santander, Spain; 22. Hospital Donostia de San Sebastían; 23. Fundación para la Formación e Investigación Sanitarias de la Región de Murcia; 24. Servicio de Neurología -Hospital de Cabueñes-Gijón; 25. Centro de Investigación y Terapias Avanzadas. Fundación CITA-alzheimer; 26. Navarrabiomed; 27. Servicio de Neurología -Hospital Universitario Central de Asturias, Oviedo; 28. Barcelona βeta Brain Research Center – Fundació Pasqual Maragall; 29. Unitat de Genètica Molecular. Institut de Biomedicina de València-CSIC; 30. Unidad Mixta de Neurologia Genètica. Instituto de Investigación Sanitaria La Fe; 31. Unitat Trastorns Cognitius, Hospital Universitari Santa Maria de Lleida, Institut de Recerca Biomédica de Lleida (IRBLLeida), Lleida, España. 32. BT-CIEN; 33. Hospital Universitario La Princesa, Madrid, Spain; 34. Hospital Clínic Barcelona).

References

1. Naj AC, Jun G, Beecham GW, Wang LS, Vardarajan BN, Buros J, Gallins PJ, Buxbaum JD, Jarvik GP, Crane PK, Larson EB, Bird TD, Boeve BF, et al. Common variants at MS4A4/MS4A6E, CD2AP, CD33 and EPHA1 are associated with late-onset Alzheimer’s disease. Nat Genet. 2011; 43:436–41.

<https://doi.org/10.1038/ng.801>
PMID:[21460841](https://pubmed.ncbi.nlm.nih.gov/21460841)

2. Proitsi P, Lupton MK, Velayudhan L, Newhouse S, Fogh I, Tsolaki M, Daniilidou M, Pritchard M, Kloszewska I, Soininen H, Mecocci P, Vellas B, Williams J, et al, Alzheimer’s Disease Neuroimaging Initiative, and GERAD1 Consortium. Genetic predisposition to increased blood cholesterol and triglyceride lipid levels and risk of Alzheimer disease: A Mendelian randomization analysis. PLoS Med. 2014; 11:e1001713.

<https://doi.org/10.1371/journal.pmed.1001713> PMID:[25226301](https://pubmed.ncbi.nlm.nih.gov/25226301)

3. Lourdusamy A, Newhouse S, Lunnon K, Proitsi P, Powell J, Hodges A, Nelson SK, Stewart A, Williams S, Kloszewska I, Mecocci P, Soininen H, Tsolaki M, et al, AddNeuroMed Consortium, and Alzheimer’s Disease Neuroimaging Initiative. Identification of cis-regulatory variation influencing protein abundance levels in human plasma. Hum Mol Genet. 2012; 21:3719–26.

<https://doi.org/10.1093/hmg/dds186> PMID:[22595970](https://pubmed.ncbi.nlm.nih.gov/22595970)

4. The Atherosclerosis Risk in Communities (ARIC) Study: design and objectives. The ARIC investigators. Am J Epidemiol. 1989; 129:687–702.

PMID:[2646917](https://pubmed.ncbi.nlm.nih.gov/2646917)

5. Psaty BM, O’Donnell CJ, Gudnason V, Lunetta KL, Folsom AR, Rotter JI, Uitterlinden AG, Harris TB, Witteman JC, Boerwinkle E, and CHARGE Consortium. Cohorts for Heart and Aging Research in Genomic Epidemiology (CHARGE) Consortium: Design of prospective meta-analyses of genome-wide association studies from 5 cohorts. Circ Cardiovasc Genet. 2009; 2:73–80.

<https://doi.org/10.1161/CIRCGENETICS.108.829747> PMID:[20031568](https://pubmed.ncbi.nlm.nih.gov/20031568)

6. Ikram MA, Seshadri S, Bis JC, Fornage M, DeStefano AL, Aulchenko YS, Debette S, Lumley T, Folsom AR, van den Herik EG, Bos MJ, Beiser A, Cushman M, et al. Genomewide association studies of stroke. N Engl J Med. 2009; 360:1718–28.

<https://doi.org/10.1056/NEJMoa0900094> PMID:[19369658](https://pubmed.ncbi.nlm.nih.gov/19369658)

7. Fried LP, Borhani NO, Enright P, Furberg CD, Gardin JM, Kronmal RA, Kuller LH, Manolio TA, Mittelmark MB, Newman A. The Cardiovascular Health Study: design and rationale. Ann Epidemiol. 1991; 1:263–76.

<https://doi.org/10.1016/1047-2797(91)90005-W> PMID:[1669507](https://pubmed.ncbi.nlm.nih.gov/1669507)

8. 3C Study Group. Vascular factors and risk of dementia: design of the Three-City Study and baseline characteristics of the study population. Neuroepidemiology. 2003; 22:316–25.

<https://doi.org/10.1159/000072920> PMID:[14598854](https://pubmed.ncbi.nlm.nih.gov/14598854)

9. Feinleib M, Kannel WB, Garrison RJ, McNamara PM, Castelli WP. The Framingham Offspring Study. Design and preliminary data. Prev Med. 1975; 4:518–25.

<https://doi.org/10.1016/0091-7435(75)90037-7> PMID:[1208363](https://pubmed.ncbi.nlm.nih.gov/1208363)

10. Splansky GL, Corey D, Yang Q, Atwood LD, Cupples LA, Benjamin EJ, D’Agostino RB Sr, Fox CS, Larson MG, Murabito JM, O’Donnell CJ, Vasan RS, Wolf PA, Levy D. The Third Generation Cohort of the National Heart, Lung, and Blood Institute’s Framingham Heart Study: design, recruitment, and initial examination. Am J Epidemiol. 2007; 165:1328–35.

<https://doi.org/10.1093/aje/kwm021> PMID:[17372189](https://pubmed.ncbi.nlm.nih.gov/17372189)

11. Filippini N, Rao A, Wetten S, Gibson RA, Borrie M, Guzman D, Kertesz A, Loy-English I, Williams J, Nichols T, Whitcher B, Matthews PM. Anatomically-distinct genetic associations of APOE epsilon4 allele load with regional cortical atrophy in Alzheimer’s disease. Neuroimage. 2009; 44:724–28.

<https://doi.org/10.1016/j.neuroimage.2008.10.003> PMID:[19013250](https://pubmed.ncbi.nlm.nih.gov/19013250)

12. Li H, Wetten S, Li L, St Jean PL, Upmanyu R, Surh L, Hosford D, Barnes MR, Briley JD, Borrie M, Coletta N, Delisle R, Dhalla D, et al. Candidate single-nucleotide polymorphisms from a genomewide association study of Alzheimer disease. Arch Neurol. 2008; 65:45–53.

<https://doi.org/10.1001/archneurol.2007.3> PMID:[17998437](https://pubmed.ncbi.nlm.nih.gov/17998437)

13. McKhann GM, Knopman DS, Chertkow H, Hyman BT, Jack CR Jr, Kawas CH, Klunk WE, Koroshetz WJ, Manly JJ, Mayeux R, Mohs RC, Morris JC, Rossor MN, et al. The diagnosis of dementia due to Alzheimer’s disease: recommendations from the National Institute on Aging-Alzheimer’s Association workgroups on diagnostic guidelines for Alzheimer’s disease. Alzheimers Dement. 2011; 7:263–69.

<https://doi.org/10.1016/j.jalz.2011.03.005> PMID:[21514250](https://pubmed.ncbi.nlm.nih.gov/21514250)

14. Moreno-Grau S, de Rojas I, Hernández I, Quintela I, Montrreal L, Alegret M, Hernández-Olasagarre B, Madrid L, González-Perez A, Maroñas O, Rosende-Roca M, Mauleón A, Vargas L, et al, GR@ACE consortium, DEGESCO consortium, and Alzheimer’s Disease Neuroimaging Initiative. Genome-wide association analysis of dementia and its clinical endophenotypes reveal novel loci associated with Alzheimer’s disease and three causality networks: The GR@ACE project. Alzheimers Dement. 2019; 15:1333–47.

<https://doi.org/10.1016/j.jalz.2019.06.4950> PMID:[31473137](https://pubmed.ncbi.nlm.nih.gov/31473137)

15. Carrasquillo MM, Zou F, Pankratz VS, Wilcox SL, Ma L, Walker LP, Younkin SG, Younkin CS, Younkin LH, Bisceglio GD, Ertekin-Taner N, Crook JE, Dickson DW, et al. Genetic variation in PCDH11X is associated with susceptibility to late-onset Alzheimer’s disease. Nat Genet. 2009; 41:192–98.

<https://doi.org/10.1038/ng.305> PMID:[19136949](https://pubmed.ncbi.nlm.nih.gov/19136949)

16. McKhann G, Drachman D, Folstein M, Katzman R, Price D, Stadlan EM. Clinical diagnosis of Alzheimer’s disease: report of the NINCDS-ADRDA Work Group under the auspices of Department of Health and Human Services Task Force on Alzheimer’s Disease. Neurology. 1984; 34:939–44.

<https://doi.org/10.1212/WNL.34.7.939> PMID:[6610841](https://pubmed.ncbi.nlm.nih.gov/6610841)

17. Gayán J, Galan JJ, González-Pérez A, Sáez ME, Martínez-Larrad MT, Zabena C, Rivero MC, Salinas A, Ramírez-Lorca R, Morón FJ, Royo JL, Moreno-Rey C, Velasco J, et al. Genetic structure of the Spanish population. BMC Genomics. 2010; 11:326.

<https://doi.org/10.1186/1471-2164-11-326> PMID:[20500880](https://pubmed.ncbi.nlm.nih.gov/20500880)

18. Antúnez C, Boada M, González-Pérez A, Gayán J, Ramírez-Lorca R, Marín J, Hernández I, Moreno-Rey C, Morón FJ, López-Arrieta J, Mauleón A, Rosende-Roca M, Noguera-Perea F, et al, and Alzheimer’s Disease Neuroimaging Initiative. The membrane-spanning 4-domains, subfamily A (MS4A) gene cluster contains a common variant associated with Alzheimer’s disease. Genome Med. 2011; 3:33.

<https://doi.org/10.1186/gm249> PMID:[21627779](https://pubmed.ncbi.nlm.nih.gov/21627779)

19. Lee JH, Cheng R, Graff-Radford N, Foroud T, Mayeux R, and National Institute on Aging Late-Onset Alzheimer’s Disease Family Study Group. Analyses of the National Institute on Aging Late-Onset Alzheimer’s Disease Family Study: implication of additional loci. Arch Neurol. 2008; 65:1518–26.

<https://doi.org/10.1001/archneur.65.11.1518> PMID:[19001172](https://pubmed.ncbi.nlm.nih.gov/19001172)

20. Bennett DA, Schneider JA, Arvanitakis Z, Wilson RS. Overview and findings from the religious orders study. Curr Alzheimer Res. 2012; 9:628–45.

<https://doi.org/10.2174/156720512801322573> PMID:[22471860](https://pubmed.ncbi.nlm.nih.gov/22471860)

21. Hofman A, Brusselle GG, Darwish Murad S, van Duijn CM, Franco OH, Goedegebure A, Ikram MA, Klaver CC, Nijsten TE, Peeters RP, Stricker BH, Tiemeier HW, Uitterlinden AG, Vernooij MW. The Rotterdam Study: 2016 objectives and design update. Eur J Epidemiol. 2015; 30:661–708.

<https://doi.org/10.1007/s10654-015-0082-x> PMID:[26386597](https://pubmed.ncbi.nlm.nih.gov/26386597)

22. Ikram MA, Brusselle G, Ghanbari M, Goedegebure A, Ikram MK, Kavousi M, Kieboom BC, Klaver CC, de Knegt RJ, Luik AI, Nijsten TE, Peeters RP, van Rooij FJ, et al. Objectives, design and main findings until 2020 from the Rotterdam Study. Eur J Epidemiol. 2020; 35:483–517.

<https://doi.org/10.1007/s10654-020-00640-5> PMID:[32367290](https://pubmed.ncbi.nlm.nih.gov/32367290)

23. Reiman EM, Webster JA, Myers AJ, Hardy J, Dunckley T, Zismann VL, Joshipura KD, Pearson JV, Hu-Lince D, Huentelman MJ, Craig DW, Coon KD, Liang WS, et al. GAB2 alleles modify Alzheimer’s risk in APOE epsilon4 carriers. Neuron. 2007; 54:713–20.

<https://doi.org/10.1016/j.neuron.2007.05.022> PMID:[17553421](https://pubmed.ncbi.nlm.nih.gov/17553421)
