# Supplementary material for: Multiomics integrative analysis identifies APOE allele-specific blood biomarkers associated to Alzheimer’s disease etiopathogenesis
Source: Aging (Albany NY). 2021 Apr 12;13(7):9277–329. doi: 10.18632/aging.202950 (PMC8064208; doi:10.18632/aging.202950)
Supplement: Supplementary Figures [file aging-13-202950-s002.pdf]

SUPPLEMENTARY FIGURES

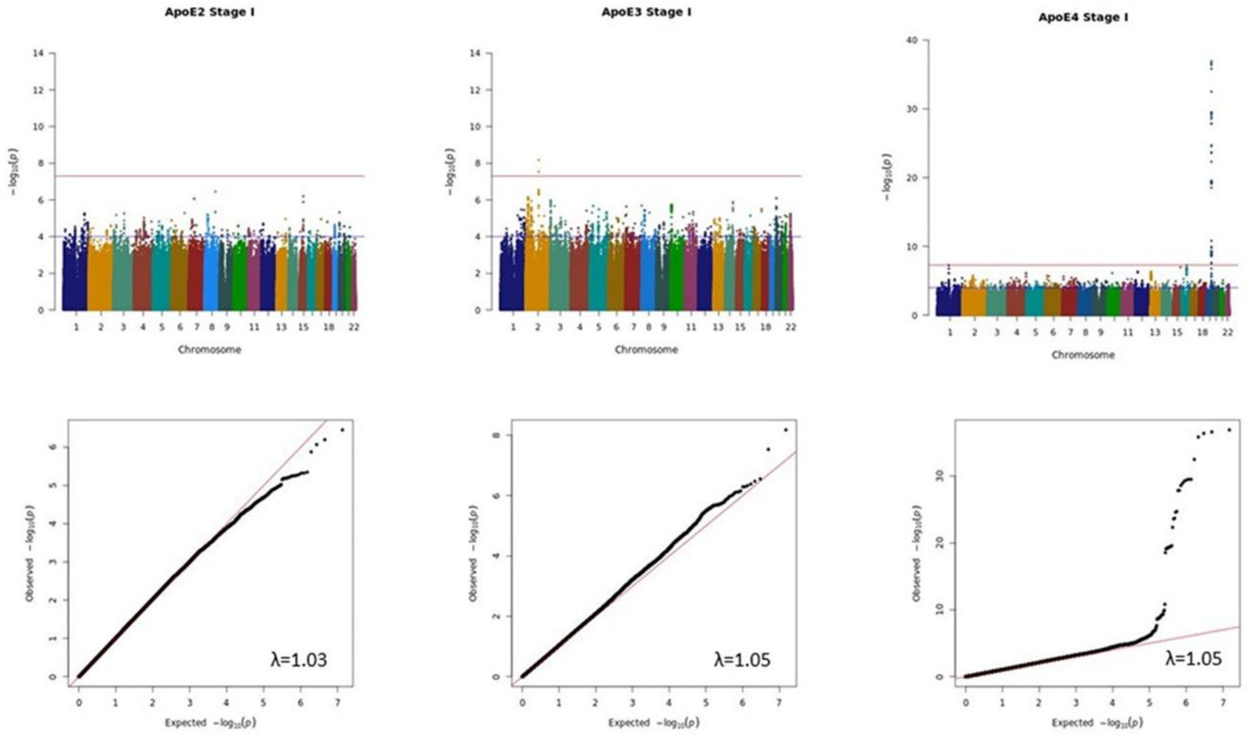

Supplementary Figure 1. Stage I meta-analysis manhattan and QQ plots (all strata).

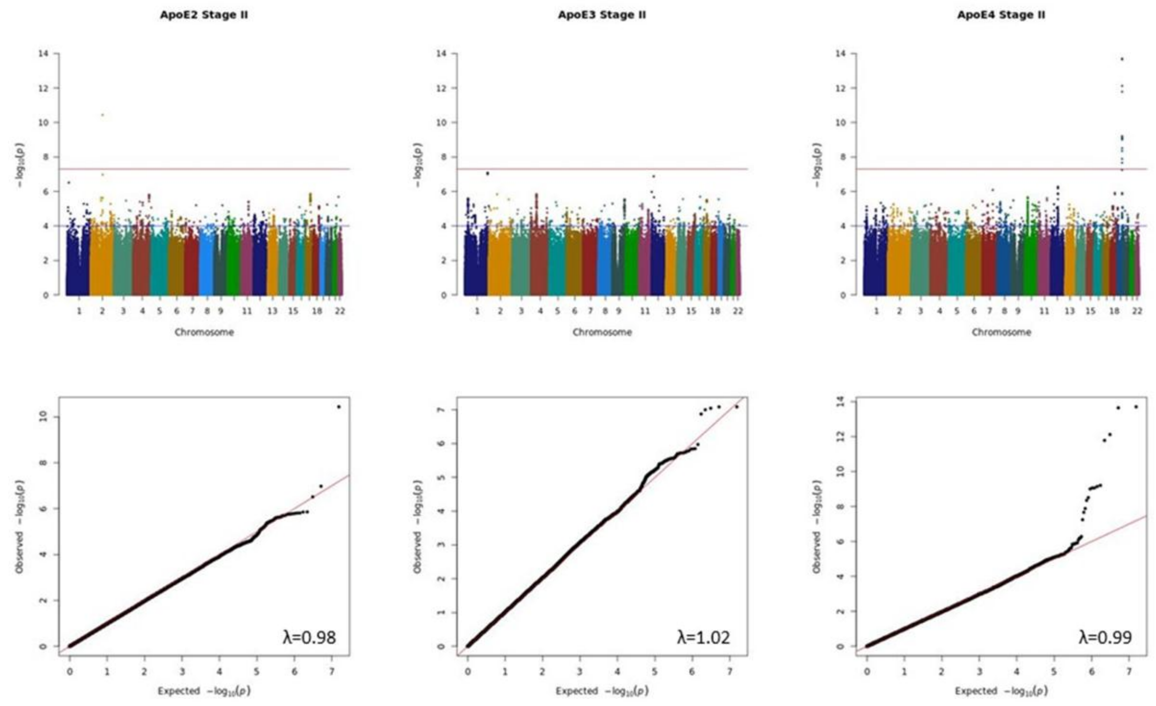

Supplementary Figure 2. Stage II meta-analysis manhattan and QQ plots (all strata).

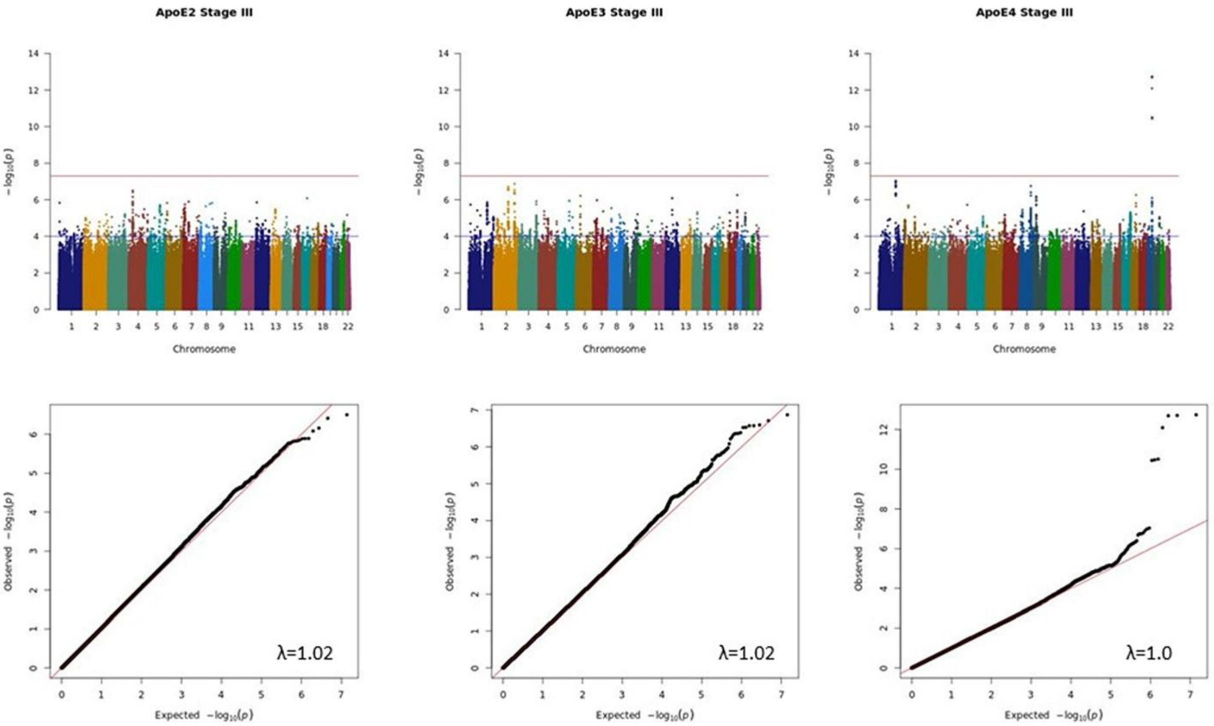

Supplementary Figure 3. Stage III meta-analysis manhattan and QQ plots (all strata).

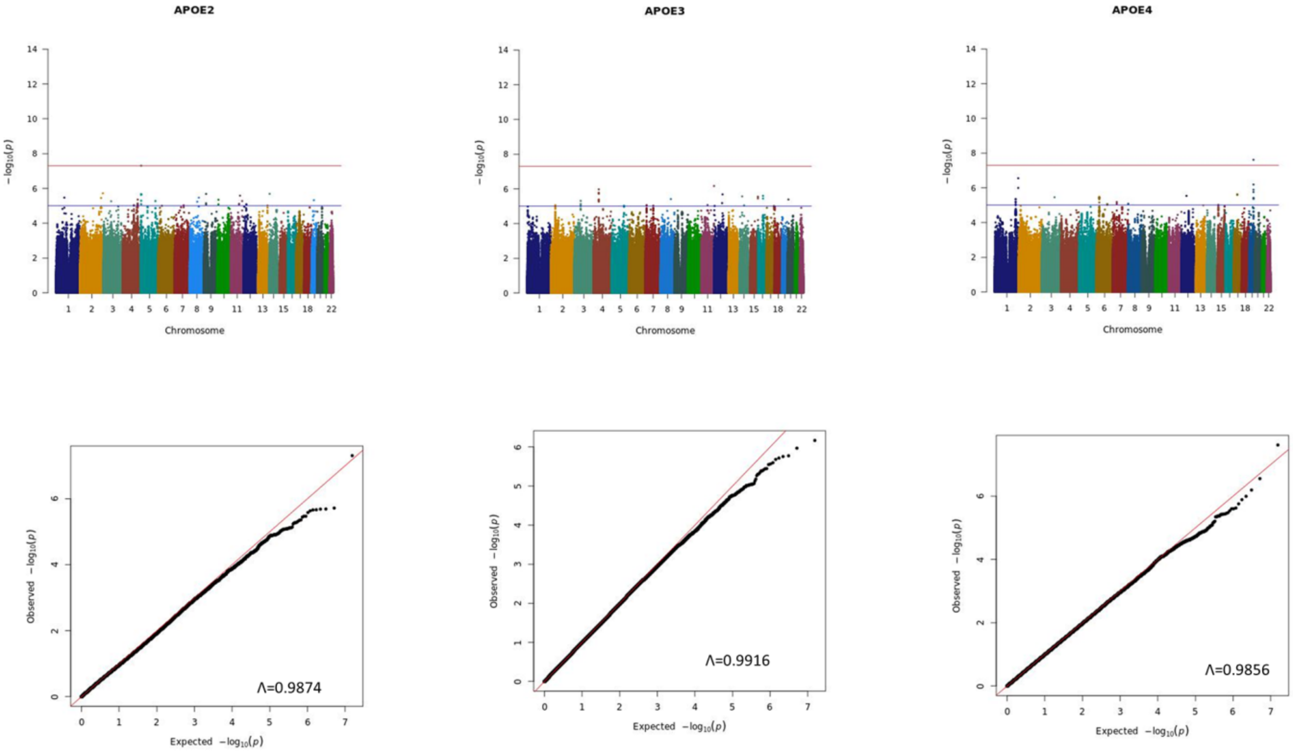

Supplementary Figure 4. Stage IV meta-analysis manhattan and QQ plots (all strata).

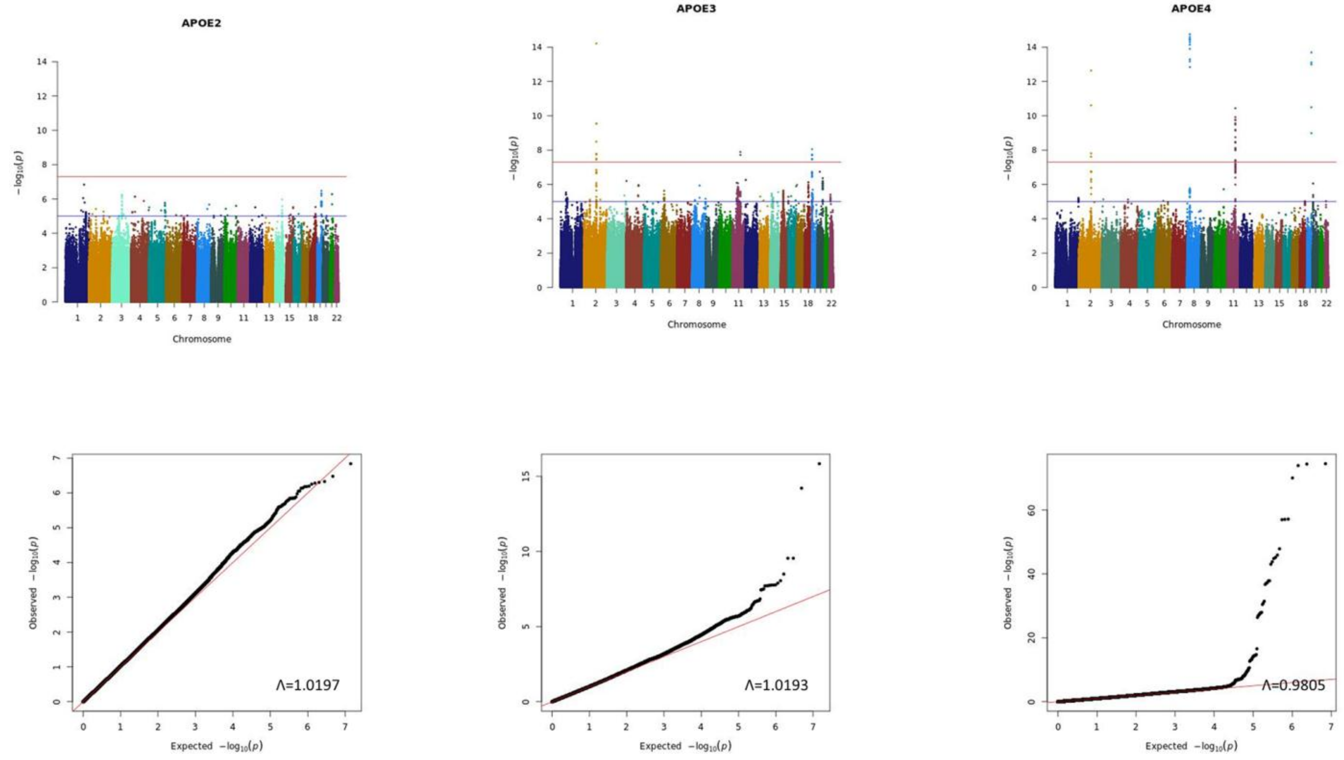

Supplementary Figure 5. Stage I-IV meta-analysis manhattan and QQ plots (all strata).

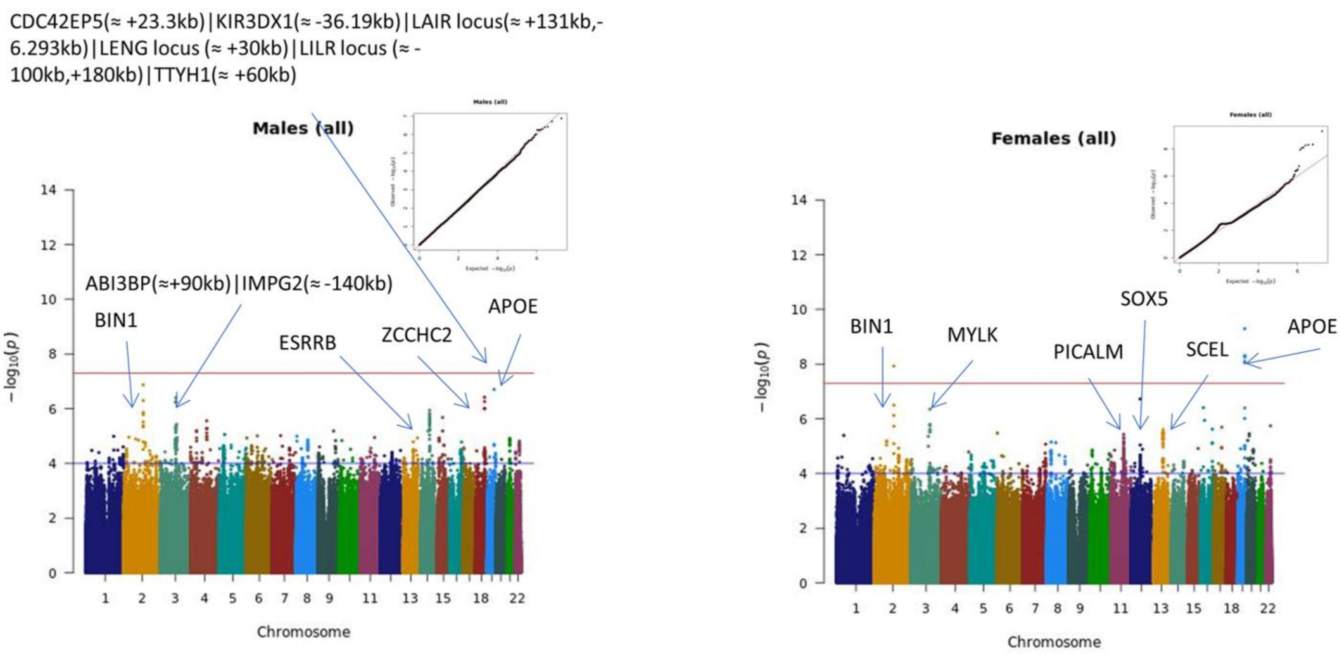

Supplementary Figure 6. Sex stratified meta-analysis manhattan and QQ plots.

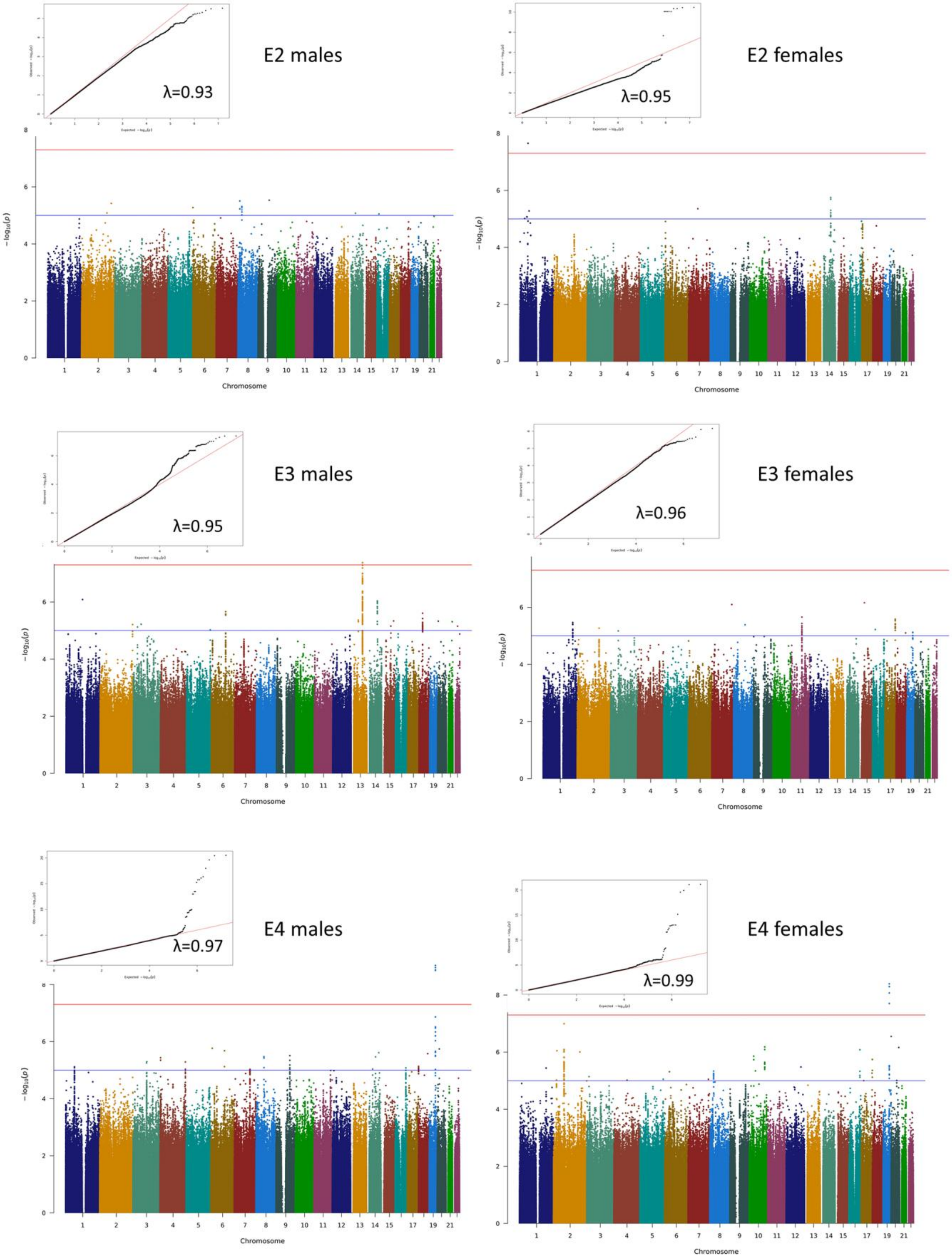

Supplementary Figure 7. APOE and sex stratified meta-analysis manhattan and QQ plots.

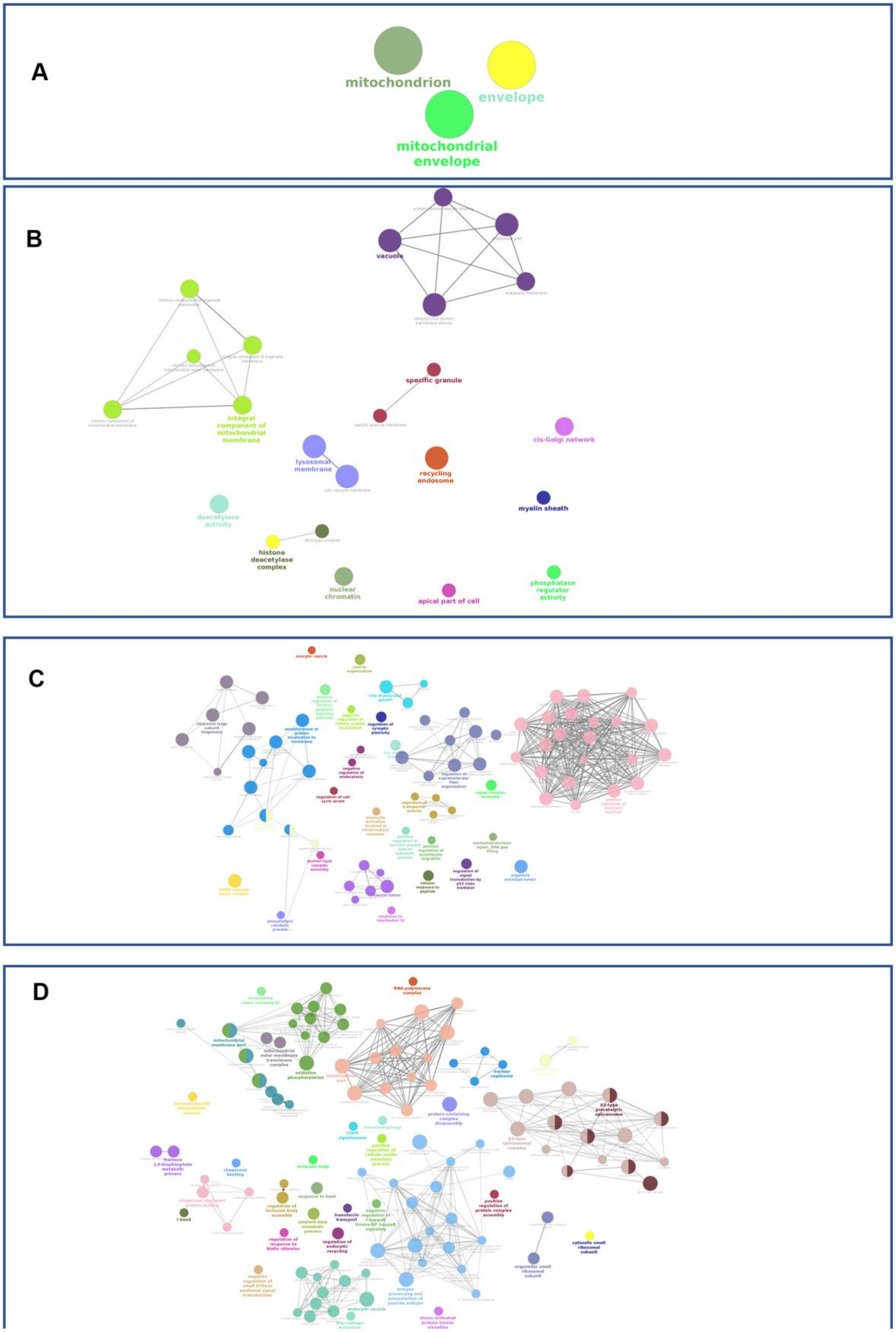

Supplementary Figure 8. RRA integrative analysis in blood: enrichment analysis for (**A**) common genes, (**B**) APOE2, (**C**) APOE3, (**D**) APOE4 specific candidates.

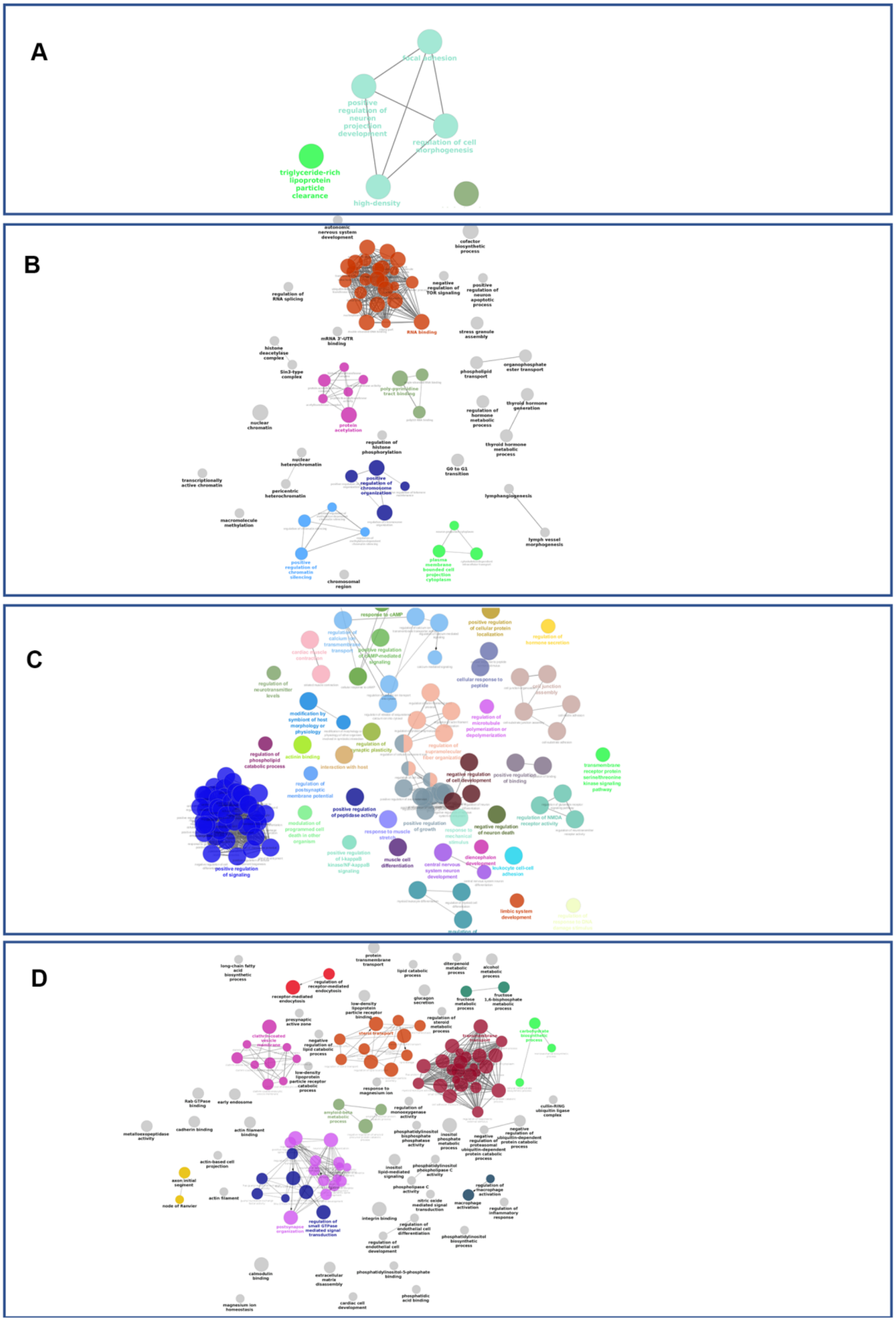

Supplementary Figure 9. RRA integrative analysis in cortex: enrichment analysis (**A**) common genes, (**B**) APOE2, (**C**) APOE3, (**D**) APOE4 specific candidates.

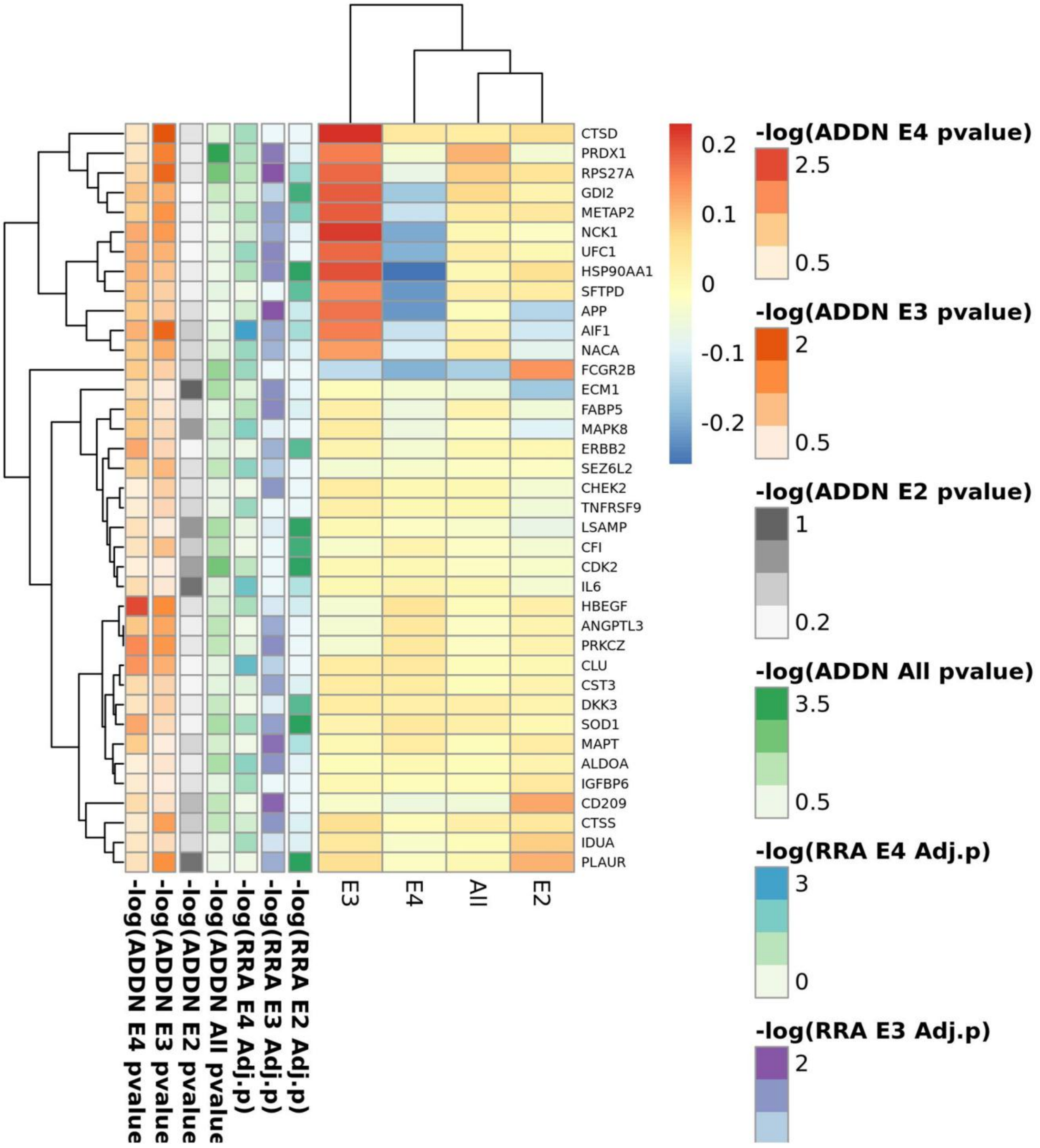

Supplementary Figure 10. Blood proteomics heatmap for RRA blood candidates (ADDN dataset, all strata).

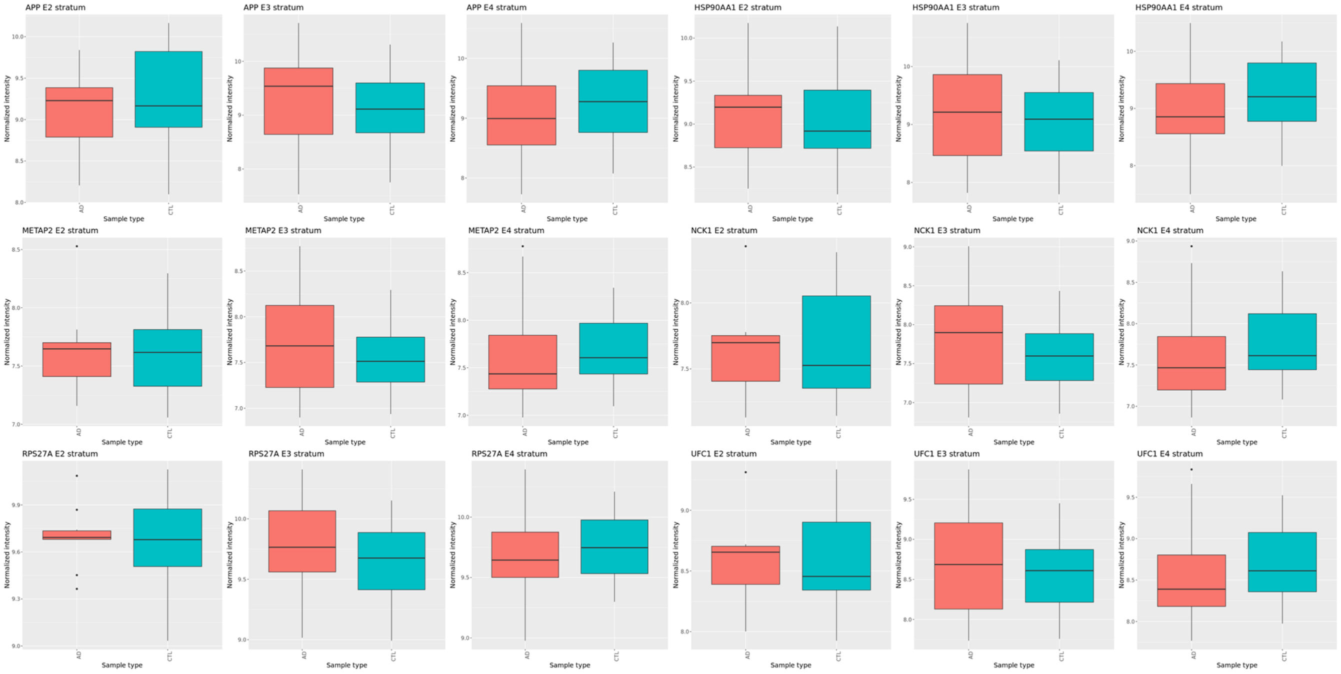

Supplementary Figure 11. Protein expression of relevant RRA blood candidates by APOE stratum and case status.

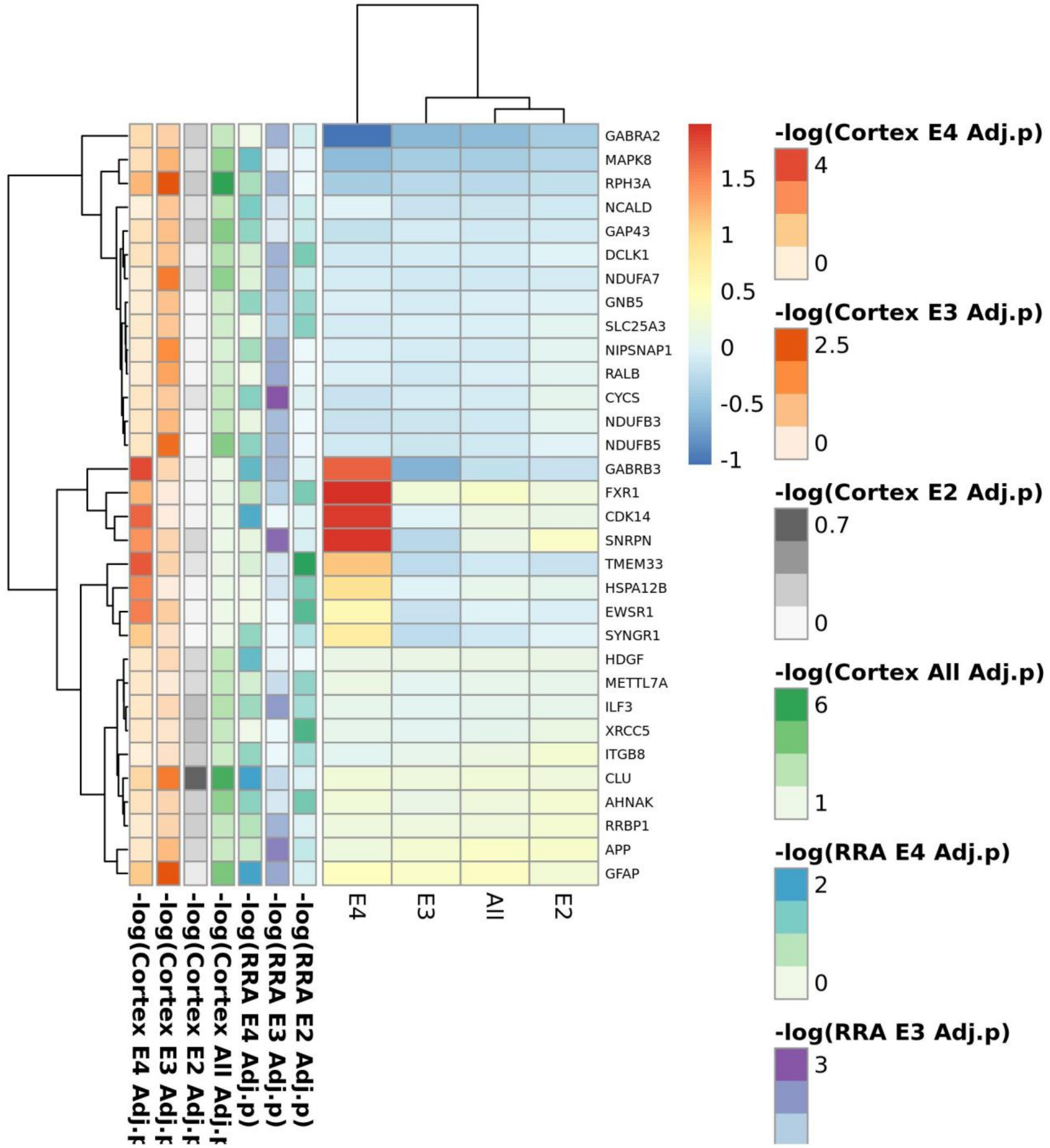

Supplementary Figure 12. Cortex proteomics heatmap for RRA cortex candidates (meta-analysis, all strata).

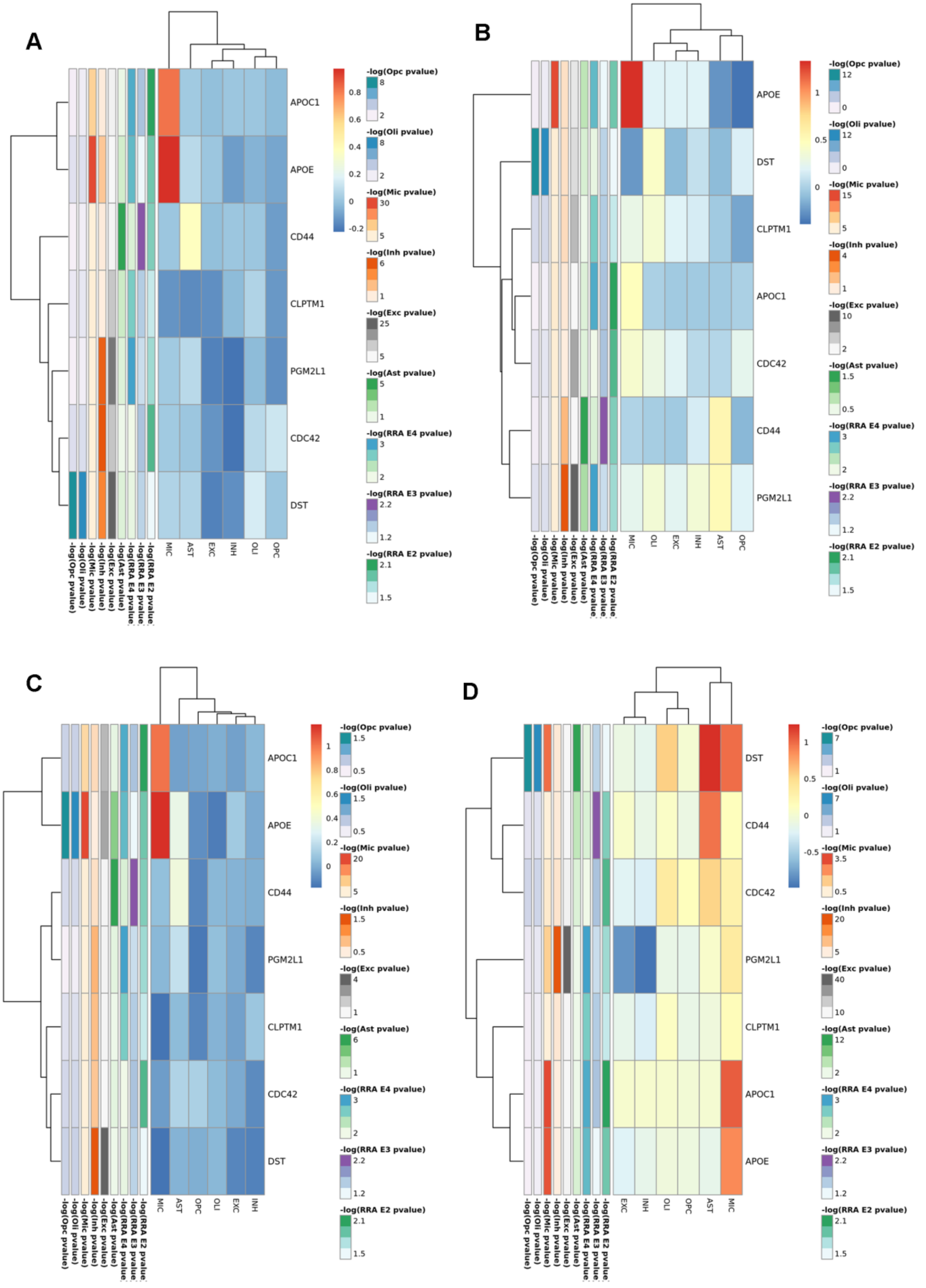

Supplementary Figure 13. Cortex snRANseq heatmap for RRA candidates shared by the three APOE strata: (**A**) Unstratified, (**B**) APOE2, (**C**) APOE3, (**D**) APOE4 AD cases vs controls.

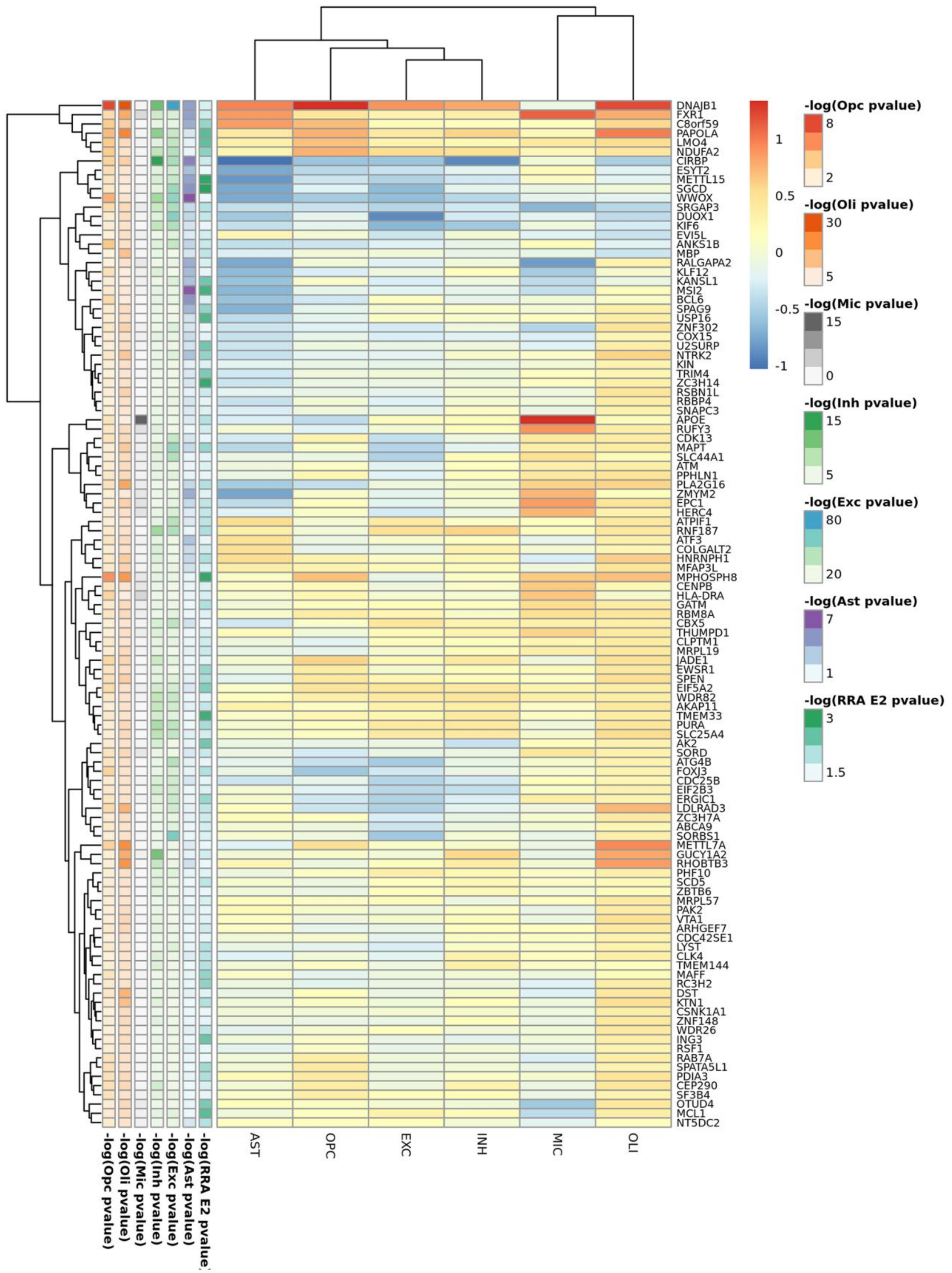

Supplementary Figure 14. Cortex snRANseq heatmap for APOE2 RRA cortex candidates.

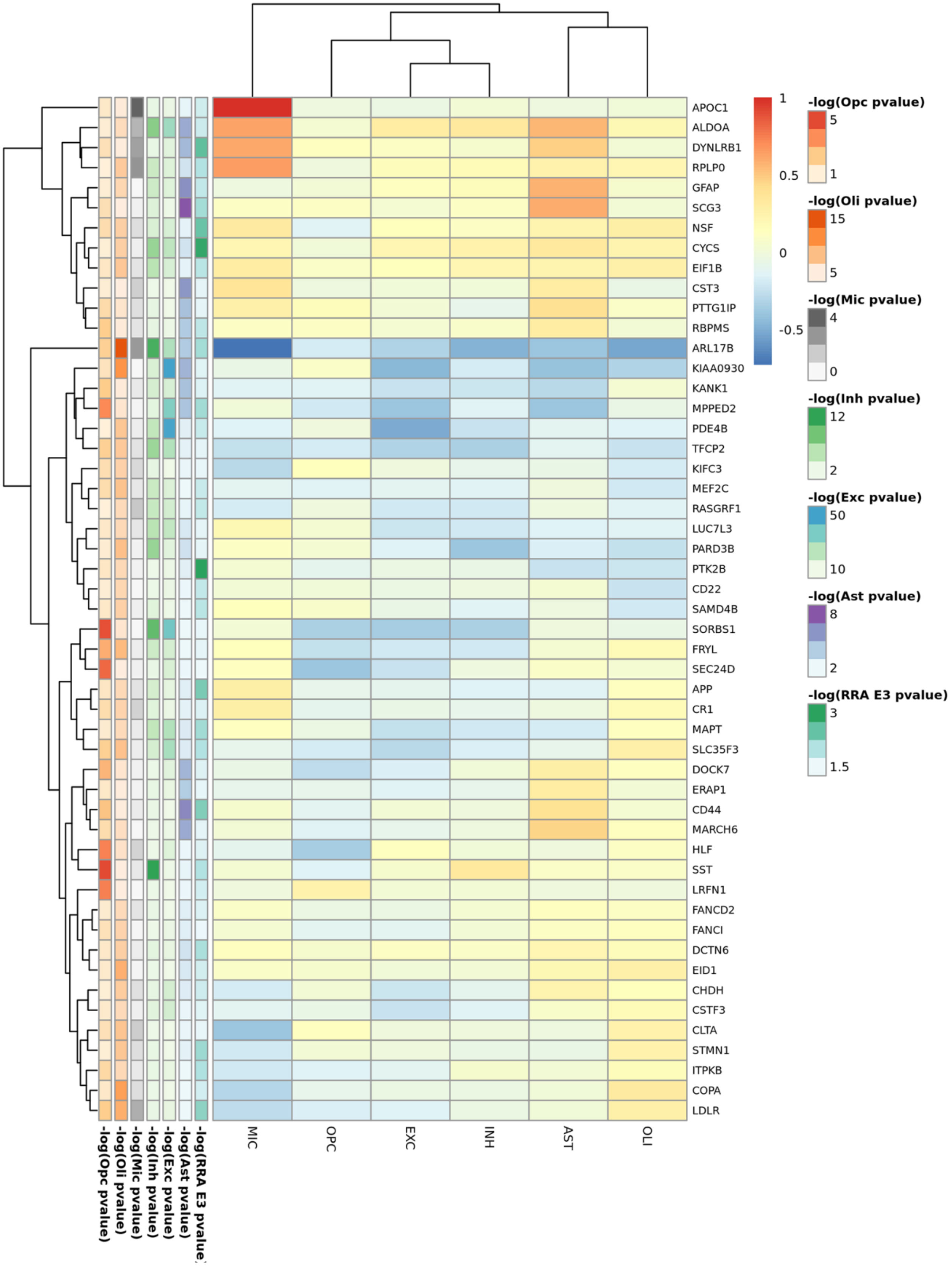

Supplementary Figure 15. Cortex snRANseq heatmap for APOE3 RRA cortex candidates.

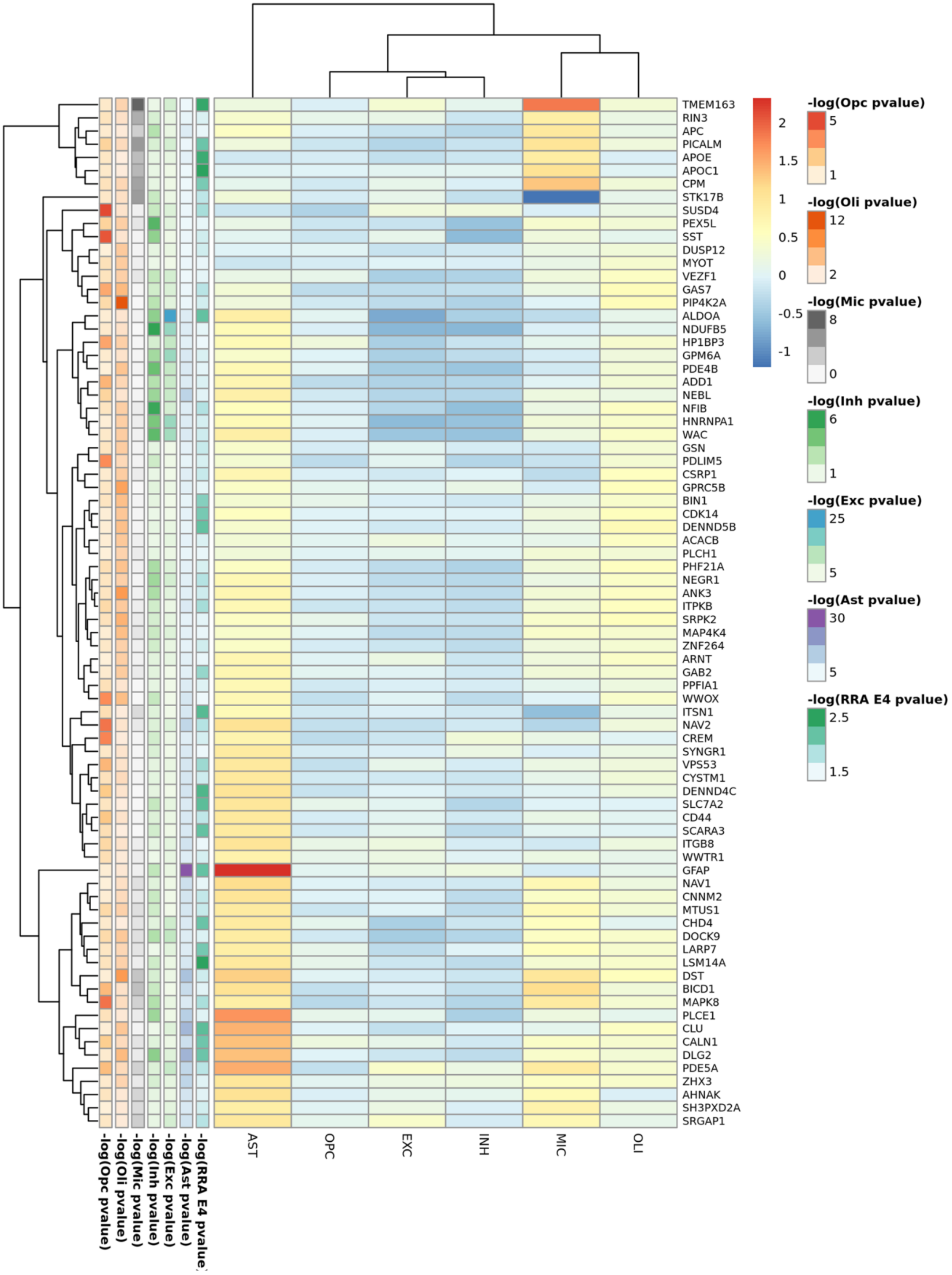

Supplementary Figure 16. Cortex snRANseq heatmap for APOE4 RRA cortex candidates.
